# Evaluation of polygenic scores for hypertrophic cardiomyopathy in the general population and across clinical settings

**DOI:** 10.1038/s41588-025-02094-5

**Published:** 2025-02-18

**Authors:** Sean L. Zheng, Sean J. Jurgens, Kathryn A. McGurk, Xiao Xu, Chris Grace, Pantazis I. Theotokis, Rachel J. Buchan, Catherine Francis, Antonio de Marvao, Lara Curran, Wenjia Bai, Chee Jian Pua, Hak Chiaw Tang, Paloma Jorda, Marjon A. van Slegtenhorst, Judith M. A. Verhagen, Andrew R. Harper, Elizabeth Ormondroyd, Calvin W. L. Chin, HCM GWAS Collaborators, Sean L. Zheng, Sean J. Jurgens, Kathryn A. McGurk, Xiao Xu, Chris Grace, Pantazis I. Theotokis, Rachel J. Buchan, Catherine Francis, Wenjia Bai, Paloma Jorda, Andrew R. Harper, Elizabeth Ormondroyd, Antonio de Marvao, Marjon A. van Slegtenhorst, James S. Ware, Antonis Pantazis, John Baksi, Brian P. Halliday, Paul Matthews, Yigal M. Pinto, Roddy Walsh, Ahmad S. Amin, Arthur A. M. Wilde, Stuart A. Cook, Sanjay K. Prasad, Paul J. R. Barton, Declan P. O’Regan, Anuj Goel, Rafik Tadros, Michelle Michels, Hugh Watkins, Connie R. Bezzina, Antonis Pantazis, John Baksi, Brian P. Halliday, Paul Matthews, Yigal M. Pinto, Roddy Walsh, Ahmad S. Amin, Arthur A. M. Wilde, Stuart A. Cook, Sanjay K. Prasad, Paul J. R. Barton, Declan P. O’Regan, R. T. Lumbers, Anuj Goel, Rafik Tadros, Michelle Michels, Hugh Watkins, Connie R. Bezzina, James S. Ware

**Affiliations:** 1 National Heart Lung Institute, Imperial College London, London, UK; 2 Medical Research Council Laboratory of Medical Sciences, Imperial College London, London, UK; 3 Royal Brompton & Harefield Hospitals, Guy’s and St. Thomas’ NHS Foundation Trust, London, UK; 4 Department of Experimental Cardiology, Amsterdam Cardiovascular Sciences, University of Amsterdam, Amsterdam UMC, Amsterdam, the Netherlands; 5 Cardiovascular Disease Initiative, Broad Institute of MIT and Harvard, Cambridge, MA USA; 6 Radcliffe Department of Medicine, University of Oxford, Division of Cardiovascular Medicine, John Radcliffe Hospital, Oxford, UK; 7 Wellcome Centre for Human Genetics, University of Oxford, Oxford, UK; 8 Department of Women and Children’s Health, King’s College London, London, UK; 9 School of Cardiovascular and Metabolic Medicine and Sciences, King’s College London, London, UK; 10 Biomedical Image Analysis Group, Department of Computing, Imperial College London, London, UK; 11 Department of Brain Sciences, Imperial College London, London, UK; 12 National Heart Research Institute Singapore, National Heart Center, Singapore, Singapore; 13 Department of Cardiology, National Heart Centre, Singapore, Singapore; 14 Cardiovascular Genetics Centre, Montreal Heart Institute, Montreal, Quebec Canada; 15 Faculty of Medicine, Université de Montréal, Montreal, Quebec Canada; 16 Department of Clinical Genetics, Erasmus MC, University Medical Center Rotterdam, Rotterdam, the Netherlands; 17 Department of Clinical Cardiology, Amsterdam Cardiovascular Sciences, University of Amsterdam, Amsterdam UMC, Amsterdam, the Netherlands; 18 European Reference Network for Rare and Low Prevalence Complex Diseases of the Heart, Paris, France; 19 Duke-National University of Singapore Medical School, Singapore, Singapore; 20 Institute of Health Informatics, University College London, London, UK; 21 Health Data Research UK London, University College London, London, UK; 22 British Heart Foundation Research Accelerator, University College London, London, UK; 23 Department of Cardiology, Thorax Center, Cardiovascular Institute, Erasmus University Medical Center, Rotterdam, the Netherlands; 24 Imperial College Healthcare NHS Trust, London, UK; 25 Program in Medical and Population Genetics, Broad Institute of MIT and Harvard, Cambridge, MA USA

**Keywords:** Cardiomyopathies, Population genetics, Personalized medicine

## Abstract

Hypertrophic cardiomyopathy (HCM) is an important cause of morbidity and mortality, with pathogenic variants found in about a third of cases. Large-scale genome-wide association studies (GWAS) demonstrate that common genetic variation contributes to HCM risk. Here we derive polygenic scores (PGS) from HCM GWAS and genetically correlated traits and test their performance in the UK Biobank, 100,000 Genomes Project, and clinical cohorts. We show that higher PGS significantly increases the risk of HCM in the general population, particularly among pathogenic variant carriers, where HCM penetrance differs 10-fold between those in the highest and lowest PGS quintiles. Among relatives of HCM probands, PGS stratifies risks of developing HCM and adverse outcomes. Finally, among HCM cases, PGS strongly predicts the risk of adverse outcomes and death. These findings support the broad utility of PGS across clinical settings, enabling tailored screening and surveillance and stratification of risk of adverse outcomes.

## Abstract (web summary)

Polygenic scores derived from genome-wide association analyses of hypertrophic cardiomyopathy and genetically correlated left ventricular traits effectively stratify risk of disease and adverse outcomes in the general population and across clinical cohorts.

## Main

Hypertrophic cardiomyopathy (HCM) is a primary cardiac disease characterized by excessive hypertrophy of the left ventricle with a population prevalence of 0.2%^1^. While many cases follow a benign course, HCM is an important cause of sudden cardiac death in young adults, and progressive disease is complicated by arrhythmia, stroke and heart failure^2,3^. Although HCM has classically been considered a Mendelian disease, a causal rare variant is identified in only one-third of cases^4,5^, with population studies highlighting the incomplete penetrance and variable expressivity of such variants^6,7^. Recent genome-wide association studies (GWAS) demonstrated that common variants contribute substantially to HCM risk (SNP, *h*^2^ = 0.29), identified many contributory loci and highlighted the complex genetic architecture of HCM^8–10^. Polygenic scores (PGS) summarize the cumulative risk arising from common variants and may provide important utility for population risk prediction and prognostication^9,11^. Still, it remains unclear whether PGS can inform the risk of HCM and clinical outcomes across broad clinical and population settings. In this study, we develop and evaluate a PGS for HCM, assessing its utility for stratification of both disease risk and severity in (1) individuals diagnosed with HCM, (2) relatives of affected individuals who are currently recommended to undergo screening and long-term surveillance, and (3) the general population, including individuals carrying disease-associated rare variants such as those that might be identified as secondary findings.

## Results

### Generation and evaluation of an HCM PGS in the general population

**Fig. 1:**
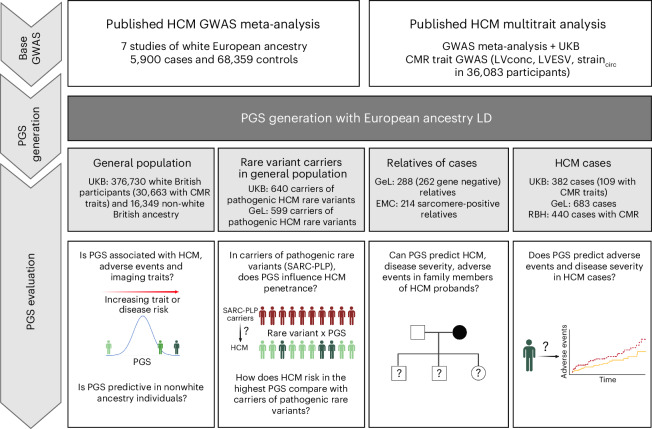
Study overview. Bayesian genome-wide PGS were generated from a published European-ancestry HCM GWAS meta-analysis of seven case–control studies (comprising 5,900 cases and 68,359 controls; PGS_GWAS_), and MTAG (analyzing HCM with three genetically correlated quantitative traits measured using CMR imaging in 36,083 European ancestry UKB participants—LV concentricity (LVconc), LEVSV and left ventricular circumferential strain (strain_circ_); PGS_MTAG_)^10^. The value of PGS to support clinical decision-making was evaluated across three key settings: in the general population (including among carriers of pathogenic rare variants in HCM-causing genes (sarcomere-positive) that might be returned as secondary findings), in relatives of HCM probands currently recommended to undergo cascade screening and surveillance, and in confirmed HCM cases under longitudinal follow-up. The figure is created with BioRender.com. SARC-PLP, pathogenic or likely pathogenic variant in sarcomeric HCM genes.

**Fig. 2:**
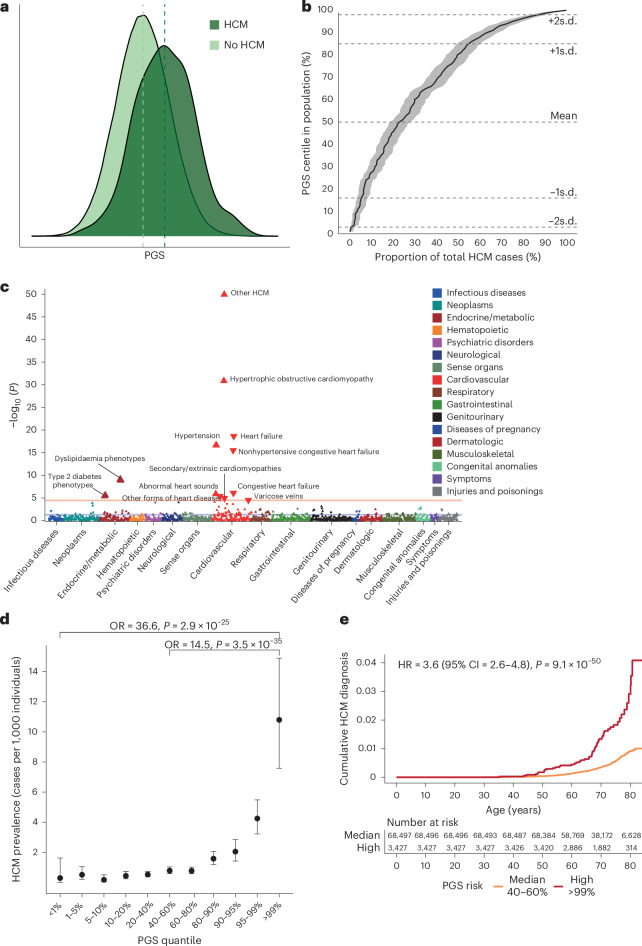
HCM PGS is associated with HCM disease status in the UKB. To validate the PGS, we analyzed associations with PGS in the UKB population. **a**, PGS_MTAG_ distribution in 374,845 UKB participants with and without HCM, demonstrating higher PGS in those with HCM. **b**, Cumulative curve of HCM cases ranked across PGS centiles. For example, approximately 75% of HCM cases have a PGS above the population 50th centile. Dashed lines represent mean, ±1 PGS s.d. and ±2 PGS s.d. Shaded line indicates 95% CI surrounding the cumulative estimate. **c**, Manhattan plot of HCM PGS phenome-wide association study in UKB, showing associations with cardiovascular and metabolic phenotypes. ICD-9 and ICD-10 diagnostic codes are mapped to Phecode Map (v1.2). Mapped phenotypes exceeding phenome-wide significance threshold (*P* = 2.7 × 10^−^^5^, red line) are labeled. Blue line indicates nominal significance (*P* < 0.05). Direction of triangle indicates the direction of effect of the PGS association. **d**, HCM prevalence and risk in UKB across the spectrum of PGS, demonstrating significantly higher HCM prevalence in individuals with the highest PGS (top centile, *n* = 3,394), compared with the median (*n* = 68,587) and lowest groups (bottom centile, *n* = 3,431). Effect estimates generated using logistic regression adjusting for age, age^2^, sex and top ten genetic principal components (PCs), with unadjusted two-sided *P* value. Data are presented as effect estimates with 95% CI. **e**, Cumulative hazards for lifetime diagnosis of HCM stratified by high (highest centile, red) and median (middle quintile, orange) PGS risk in UKB. HR calculated using Cox proportional hazards model, adjusted for age, age^2^, sex and first ten genetic PCs, with two-sided *P* value.

PGS was generated using the largest published GWAS comprising a total of 5,900 unrelated HCM cases and 68,359 controls of European ancestry from seven cohorts (PGS_GWAS_), and multitrait analysis of GWAS (MTAG) incorporating the HCM GWAS with GWAS of three genetically correlated cardiac magnetic resonance (CMR) imaging traits (left ventricular concentricity, left ventricular end-systolic volume (LVESV) and left ventricular circumferential strain) in 36,083 European ancestry participants in the UK Biobank^10^ (UKB; PGS_MTAG_; Fig. 1). In an independent cohort of 343,182 unrelated white British ancestry participants in UKB, PGS_MTAG_ was associated with the risk of HCM (defined using International Classification of Diseases 9 and 10 (ICD-9 and ICD-10) codes, self-reporting and/or CMR imaging; Supplementary Note; odds ratio (OR) = 2.34 per PGS_MTAG_ s.d., 95% confidence interval (CI) = 2.12–2.59, *P* < 2 × 10^−^^16^), provided better predictive performance than PGS_GWAS_ (OR = 1.97 per PGS_GWAS_ s.d., 95% CI = 1.81–2.15, *P* < 2 × 10^−^^16^; Supplementary Table 1) and was therefore used for all subsequent analyses unless otherwise stated (Supplementary Fig. 1). The distribution of PGS in the UKB population-based cohort is shown in Fig. 2a. Among HCM cases, 75.1% (95% CI = 71.4–79.5) have a PGS above the population mean, while those with a PGS greater than one standard deviation above the mean accounted for 46.4% (95% CI = 41.2–51.7) of cases (Fig. 2b). Having demonstrated associations between PGS and HCM risk, we evaluated effect sizes in the general population. Individuals with PGS in the highest centile (prevalence = 10.9 cases per 1,000 individuals) had significantly higher risks of HCM compared with those in the median (prevalence = 0.8 cases per 1,000 individuals, OR = 14.5, 95% CI = 9.5–22.2, *P* = 3.5 × 10^−^^35^; time to HCM diagnosis—hazards ratio (HR) = 3.6, 95% CI = 2.6–4.8, *P* < 2 × 10^−^^16^) and lowest centiles (prevalence = 0.3 cases per 1,000 individuals, OR = 36.6, 95% CI = 18.6–72.2, *P* = 2.9 × 10^−^^25^; Fig. 2c,d, Supplementary Fig. 2 and Supplementary Tables 2 and 3).

Exploring the role of polygenic risk on the expressivity of an HCM phenotype in 30,663 white British ancestry UKB participants who underwent CMR, PGS_GWAS_ was associated with traits that are classically seen in HCM^6^: increased cardiac hypertrophy (maximum left ventricular wall thickness (maxLVWT) = +0.13 mm per PGS s.d., *P* = 1.1 × 10^−^^80^; highest versus lowest PGS centile = 9.8 versus 9.1 mm, *P* = 9 × 10^−^^9^), increased cardiac contractility (left ventricular ejection fraction (LVEF) = +0.6%, *P* = 2.7 × 10^−^^64^; 61.3% versus 57.7%, *P* = 2.7 × 10^−^^13^), reduced chamber volumes (left ventricular end-diastolic volume (LVEDV) = −2.0 ml, *P* = 1.2 × 10^−^^46^; 142.1 versus 154.9 ml, *P* = 8.3 × 10^−^^8^) and LVESV (−1.7 ml, *P* = 6.9 × 10^−^^80^; 55.9 versus 66.4 ml, *P* = 2.7 × 10^−^^10^), all of which were biventricular in nature (Supplementary Fig. 3 and Supplementary Tables 4 and 5), and persisted when excluding participants with HCM (Supplementary Table 6).

Phenome-wide association study (PheWAS) of 1,839 clinical diagnoses in the UKB identified PGS associations with hypertension and metabolic phenotypes (dyslipidemia and type 2 diabetes; Supplementary Table 7). Mendelian randomization (MR) highlighted causal influence of blood pressure and body mass index, albeit with evidence of significant pleiotropy^8^, and no significant associations with lipid and glycemic traits (Supplementary Table 8 and Supplementary Fig. 4). The inverse association with heart failure^9^ (Fig. 2e) and absence of expected associations (for example, atrial fibrillation or flutter^4^) may be explained by the reciprocal relationship of HCM common genetic risk with dilated cardiomyopathy (DCM)^9^ (UKB DCM—OR = 0.88 per PGS decile, 95% CI = 0.85–0.90, *P* < 2 × 10^−^^16^; OR = 0.69 per PGS s.d., 95% CI = 0.64–0.74; Supplementary Fig. 2). MR analysis identified causal protective associations with heart failure (MR inverse variance weighted (IVW) *β* = −0.09, *P* = 1.3 × 10^−^^3^). For atrial fibrillation, MR analysis showed no overall causal relationship (MR IVW *β* = 0.02, *P* = 0.39), with individual HCM-risk variants associated with both increased and decreased atrial fibrillation risk, highlighting the complex pleiotropic relationship (Supplementary Fig. 5).

### PGS performance in non-European ancestry populations

PGS derived from one ancestry underperform when applied to different or more diverse ancestral populations^12–15^. We adapted the European-ancestry PGS (PGS_GWAS_) by applying ancestry-specific linkage disequilibrium references^16^ and evaluated its performance for HCM status in 16,349 UKB participants of non-European ancestry (7,542 South Asian, 7,348 African and 1,457 East Asian ancestry), and for CMR quantitative traits in a subset. PGS distributions differed between the different ancestry groups (ANOVA *P* < 2 × 10^−^^16^, Tukey adjusted *P* < 2.5 × 10^−^^8^ for between-group comparisons), with PGS highest in African ancestry (HCM prevalence = 0.4%), and lowest in South Asian ancestry (HCM prevalence = 0.1%; Supplementary Fig. 6). Although analysis within each ancestral group was limited by power, as expected, PGS performance appeared to be poorer (South Asian (9 HCM cases, OR = 1.82 per PGS s.d., *P* = 0.068), African (27 cases, OR = 1.21 per PGS s.d., *P* = 0.35) and insufficient East Asian ancestry cases to allow estimation (2 cases)) compared within white British (OR = 2.34 per PGS s.d., *P* < 2 × 10^−^^16^; Supplementary Fig. 6 and Supplementary Table 9).

To improve cross-ancestral polygenic prediction, we performed GWAS in an unrelated East Asian ancestry cohort of 174 HCM cases and 776 controls recruited from Singapore (no individual SNPs reaching genome-wide significance, Supplementary Fig. 7), and combined them with PGS_GWAS_ to generate a cross-population PGS (PGS_East Asian_) using PRS-CSx^16^. In 111 East Asian ancestry individuals with CMR in the UKB, PGS_East Asian_ was nominally associated with left ventricular volumetric (LVESV, change per PGS s.d. = −3.6 ml, *P* = 0.025, *P*_adj_ = 0.11) and wall thickness (maxLVWT = +0.28 mm, *P* = 0.017, *P*_adj_ = 0.09) traits, which were not present when using the European only PGS_GWAS_ (Supplementary Fig. 6 and Supplementary Table 10). This suggests that even a modest ancestry-specific GWAS can improve performance when applying a PGS in a new population.

### PGS modulates the penetrance of HCM-causing rare variants

**Fig. 3:**
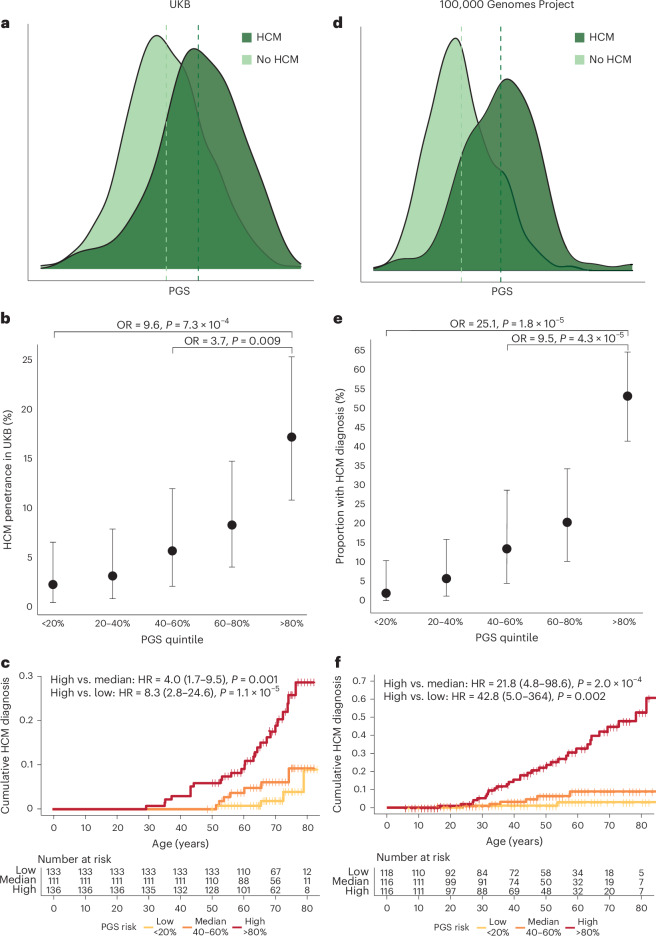
PGS modulates HCM penetrance in carriers of rare pathogenic variants in HCM-associated genes. **a**–**c**, UKB represents a broadly unselected population, as participants were not recruited based on genotype or phenotype. **d**–**f**, 100,000 Genomes Project (GeL) comprises a mix of participants recruited based on cardiomyopathy and participants recruited with other rare diseases, cancer or as relatives of patients with a rare disease. **a**,**d**, The PGS distribution is shown in 640 sarcomere-positive UKB participants (**a**) and 599 GeL participants (**d**) with and without HCM, validating that PGS is higher in cases than controls. **b**,**e**, Among sarcomere-positive individuals, the highest PGS quintile (UKB, *n* = 136; GeL, *n* = 116) was associated with increased HCM diagnosis compared with median (UKB, *n* = 111; GeL, *n* = 116) and lowest quintiles (UKB, *n* = 133; GeL, *n* = 118). Effect estimates generated using logistic regression adjusting for age, age^2^, sex and top ten genetic PCs, with unadjusted two-sided *P* value. Data are presented as effect estimates with 95% CI. **c**,**f**, The time to HCM diagnosis in highest, median and lowest quintiles, shows that those with higher PGS are at increased risk of HCM, and develop disease earlier, which is important for lifetime burden of disease morbidities. HR calculated using Cox proportional hazards model, adjusted for age, age^2^, sex and first ten genetic PCs, with two-sided *P* value.

Among 318,945 UKB participants with whole-exome sequencing (WES), 640 were unrelated carriers of pathogenic or likely pathogenic variants in eight genes encoding components of the cardiac sarcomere (*MYBPC3*, *MYH7*, *TNNT2*, *TNNI3*, *TPM1*, *ACTC1*, *MYL3* and *MYL2*; ‘sarcomere-positive’)^17^. A total of 336 HCM cases were identified, of which 43 cases were sarcomere-positive (penetrance = 6.7%, 95% CI = 4.9–8.9) and 293 cases were sarcomere-negative (prevalence = 0.09%, 95% CI = 0.08–0.1). Among the UKB population, there was no difference in mean PGS in sarcomere-positive and sarcomere-negative participants (*P* = 0.60; Supplementary Fig. 8), arguing against any unmeasured relationship between rare sarcomeric variants and common variant polygenicity that might arise from selective ascertainment. PGS was associated with HCM in both sarcomere-positive (OR = 2.35 per PGS s.d., *P* = 1.1 × 10^−^^6^) and sarcomere-negative participants (OR = 2.15 per PGS s.d., *P* < 2 × 10^−^^16^; Fig. 3a). Among the 640 unrelated sarcomere-positive individuals in UKB, penetrance by middle-older age (median age = 72 years, IQR ± 13 years) was markedly greater in those in the highest PGS quintile (HCM penetrance = 17.2%, 95% CI = 10.8–25.3) when compared with the median (5.7%, 95% CI = 2.1–12.0; highest versus median quintile—OR = 3.69, 95% CI = 1.46–10.67, *P* = 0.009) and lowest quintiles (2.3%, 95% CI = 0.5–6.6; highest versus lowest quintile—OR = 9.56, 95% CI = 2.95–43.89, *P* = 7.3 × 10^−^^4^; Fig. 3b). In time-to-event analyses, the risk of HCM diagnosis (HR = 3.98, 95% CI = 1.66–9.52, *P* = 0.001) and adverse HCM outcomes (HR = 1.56, 95% CI = 1.1–2.34, *P* = 0.029) were similarly greater in the highest compared with median quintile (Fig. 3c and Supplementary Fig. 8).

We confirmed the modulatory role of PGS on rare variants in 100,000 Genomes Project (GeL)^18^ (Fig. 3d) a study that recruited individuals with rare diseases (including cardiomyopathies) and their relatives. Because a small proportion of the GeL cohort was ascertained based on cardiomyopathy, we cannot use these data to directly quantify penetrance, but can nonetheless assess the effect size for PGS in combination with a rare variant on disease risk in this cohort. There were 599 sarcomere-positive participants, of which 72 were HCM cases (proportion affected = 12.0%, 95% CI = 9.6–15.0%). PGS_GeL_ (generated from MTAG summary statistics leaving out the GeL cohort) was associated with prevalent HCM (OR = 3.53 per PGS_GeL_ s.d., 95% CI = 2.59–4.80, *P* = 9.8 × 10^−^^16^; OR = 1.60 per PGS_GeL_ decile, 95% CI = 1.41–1.85). Sarcomere-positive individuals with high PGS_GeL_ (highest quintile) were more than 9 times as likely to have been ascertained as cases compared with the median (OR = 9.50, 95% CI = 3.60–32.6, *P* = 4.3 × 10^−^^5^), and 25 times as likely compared with the lowest quintiles (OR = 25.1, 95% CI = 7.3–160.3, *P* = 1.8 × 10^−^^5^; Fig. 3e). The hazards of HCM diagnosis was higher in the highest quintile than at the median (HR = 21.8, 95% CI = 4.8–98.6, *P* = 0.0002) and lowest quintiles (HR = 42.8, 95% CI = 5.0–364.0, *P* = 0.002; Fig. 3f). Finally, of 527 sarcomere-positive individuals without a diagnosis of HCM on recruitment to the 100,000 Genomes Project, a total of 7 were diagnosed with HCM on follow-up, 5 of whom had PGS_GeL_ in the highest quintile.

### Pathogenic rare variant effects remain greater than PGS risk

It has been suggested for several diseases that extreme PGS risk confers a similar magnitude of increased risk as the presence of Mendelian pathogenic variants (for example, familial hypercholesterolemia for coronary artery disease, and *BRCA1* or *BRCA2* for breast cancer^12,19,20^). In the UKB, while sarcomere-negative individuals with PGS at the uppermost extreme (defined as the top 0.25%—a frequency comparable to population estimates of pathogenic HCM rare variants (1 in 400)^6^) had an 18-fold increased risk of having HCM compared with the median (OR = 18.1, 95% CI = 10.0–32.9, *P* = 1.6 × 10^−^^21^), they were at significantly lower risk of HCM and severity of imaging traits compared with sarcomere-positive individuals (OR = 5.4, 95% CI = 2.8–11.6, *P* = 2.2 × 10^−^^8^; Supplementary Table 11). These findings suggest that while PGS accounts for an important component of risk in sarcomere-negative individuals, genetic HCM risk is highest among carriers of rare pathogenic variants in sarcomeric genes.

### PGS stratifies the risk of HCM in relatives of probands

Understanding the penetrance of HCM in relatives of probands will have important implications on clinical practice (for example, screening and longitudinal surveillance). We sought to assess whether PGS modulates penetrance in relatives of sarcomere-positive HCM cases, and stratifies risk in sarcomere-negative families, in two cohorts.

**Fig. 4:**
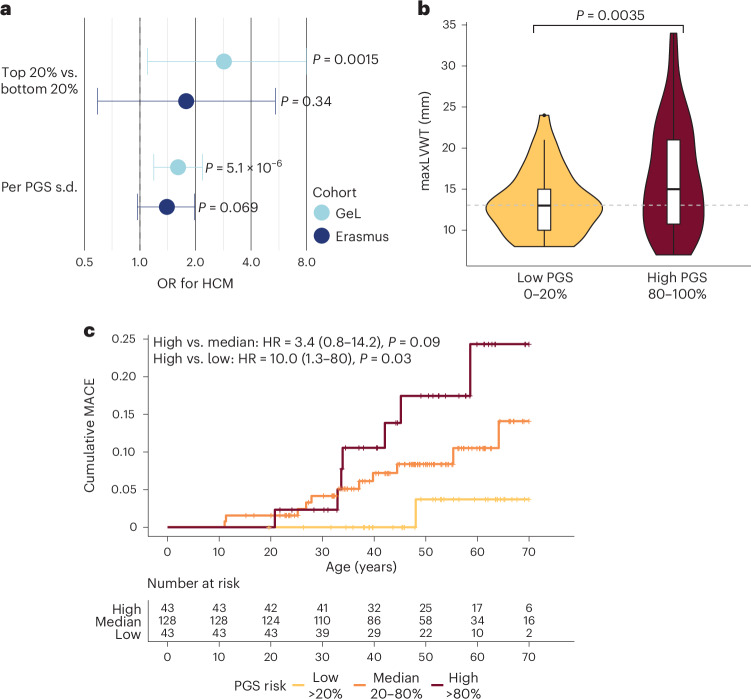
PGS associate with HCM risk and adverse outcomes in relatives of HCM cases. To evaluate applications of PGS in families undergoing screening and surveillance for HCM, we studied the relatives of HCM cases in two cohorts, GeL and EMC cohort. **a**, OR for HCM among relatives of HCM probands in the two cohorts (GeL, *n* = 288; EMC, *n* = 214), stratified by PGS. **b**, Violin and box and whisker plot of maxLVWT in sarcomere-positive relatives stratified by highest (*n* = 40) and lowest (*n* = 38) PGS_EMC_ quintiles. Box plot indicate median and interquartile range, whiskers denote 1.5× the interquartile range, outliers shown separately, and the edges of violin plots indicate minimum and maximum values. Dashed line indicates a 13-mm cutoff used for guideline diagnosis of HCM in relatives of individuals with HCM. **c**, Cumulative major adverse cardiovascular events (MACE) among 214 sarcomere-positive relatives of HCM index patients stratified by PGS_EMC_ above or below the median. MACE was defined as a composite of septal reduction therapy, cardiac transplantation, aborted cardiac arrest, appropriate defibrillator shock or sudden cardiac death. To avoid inflation of PGS performance resulting from sample overlap, PGS were rederived from GWAS leaving out the cohort that the PGS was being evaluated in (GeL–PGS_GeL_, EMC–PGS_EMC_). HR calculated using Cox proportional hazards model, adjusted for sex, first four genetic PCs, and genetic relatedness matrix, with two-sided *P* value.

The 100,000 Genomes Project was initially designed to evaluate genetically unexplained rare disease through the recruitment of cases and their relatives, and therefore, the cohort has a higher proportion of genetically unexplained sarcomere-negative cases than in the clinical setting (pathogenic rare variants were identified in 94 out of 919 HCM cases). In all, 288 relatives of 193 HCM index cases (262 gene-negative, and 26 gene-positive relatives of 14 gene-positive HCM cases) were recruited, of whom 116 had prevalent HCM and 6 were diagnosed with HCM during follow-up. PGS_GeL_ was higher in probands (*P* < 2 × 10^−^^16^) and affected relatives (*P* = 3.6 × 10^−^^6^) compared with unaffected relatives, with no difference between probands and affected relatives (*P* = 0.99). PGS was associated with increased risk of HCM (HCM—OR = 1.74 per PGS_GeL_ s.d., 95% CI = 1.37–2.21, *P* = 5.1 × 10^−^^6^; highest versus median quintile—OR = 4.17, 95% CI = 1.78–10.5, *P* = 0.0015; Fig. 4a). Of 178 relatives who did not have a diagnosis of HCM on recruitment, 6 were diagnosed on follow-up (mean = 5.1 years), all with PGS in the highest quintile.

The Erasmus Medical Center (EMC) cohort comprises 214 relatives of 184 index HCM cases, all carriers of rare pathogenic variants in sarcomere-encoding genes. After clinical evaluation, 135 relatives were found to have HCM. Although the PGS_EMC_ (derived using HCM MTAG omitting EMC cohort) was not significantly associated with HCM in relatives (OR = 1.4 per PGS_EMC_ s.d., 95% CI = 0.97–1.98, *P* = 0.069; Fig. 4a), it was associated with increased maxLVWT (+1.4 mm per PGS_EMC_ s.d., 95% CI = 0.6–2.1, *P* = 5.0 × 10^−^^4^; highest versus lowest quintile—+3.5 mm, 95% CI = 1.26–6.41, *P* = 0.0035) and, importantly, with increased risk of major adverse cardiovascular events after study enrollment (HR = 1.74 per PGS_EMC_ s.d., 95% CI = 1.03–2.91, *P* = 0.036; highest versus lowest quintile—HR = 17.7, 95% CI = 0.9–347, *P* = 0.058), prevention of which is the primary motivation for cascade screening and early diagnosis (Fig. 4b,c and Supplementary Fig. 9).

### PGS as a new prognostic marker in HCM

**Fig. 5:**
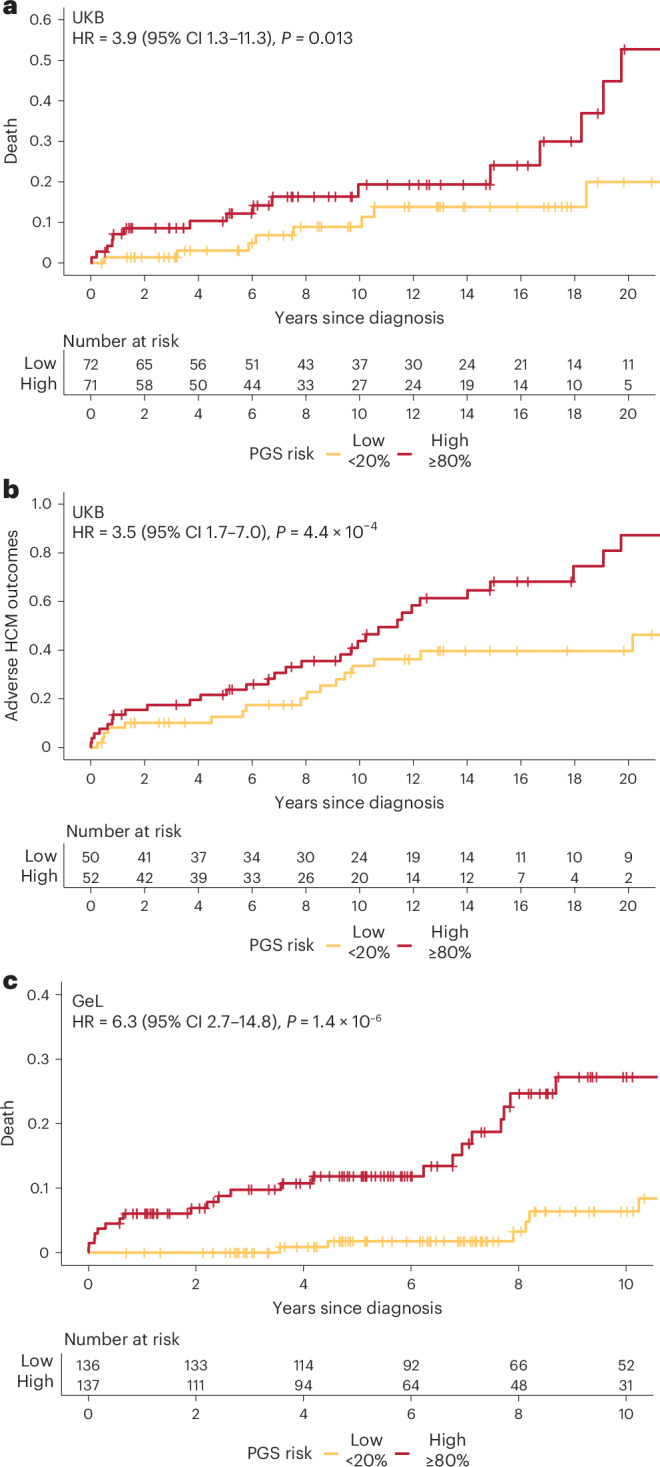
PGS stratifies the risk of death and adverse outcomes in individuals with HCM. **a**–**c**, Cumulative all-cause mortality and adverse HCM outcomes after HCM diagnosis in 382 HCM cases from the UKB (**a**,**b**) and cumulative all-cause mortality in 683 HCM cases from GeL (**c**), stratified by PGS in the highest and lowest quintiles. Adverse HCM outcomes include death, stroke, cardiac arrest, implantable cardioverter-defibrillator implantation, septal reduction therapy (alcohol–septal ablation or surgical myectomy), LVAD implantation or cardiac transplantation. HR calculated using Cox proportional hazards model, adjusted for age, age^2^, sex and first ten genetic PCs, with two-sided *P* value.

Although some individuals with HCM have a relatively benign disease course, an important proportion suffer adverse outcomes, including cardiovascular death, and risk stratification, especially for preventable sudden death, remains an urgent clinical need. We sought to investigate whether PGS was associated with adverse outcomes and clinical features of severity in individuals with HCM. In 382 HCM cases in the UKB, a PGS in the highest quintile was associated with an increased risk of death and adverse cardiovascular outcomes after HCM diagnosis (death—highest versus lowest quintile, HR = 3.88, 95% CI = 1.33–11.29, *P* = 0.013; adverse outcomes (HCM composite)—HR = 3.50, 95% CI = 1.74–7.03, *P* = 4.4 × 10^−^^4^; Fig. 5a,b, Supplementary Fig. 10 and Supplementary Table 12). In 683 HCM cases in GeL, cases in the highest quintile had a sixfold increased risk of death after HCM diagnosis (HR = 6.30, 95% CI = 2.68–14.78, *P* = 1.4 × 10^−^^6^; Fig. 5c and Supplementary Fig. 10). In 101 sarcomere-positive HCM cases from a clinical cohort (Royal Brompton & Harefield Hospitals, RBH), higher PGS was associated with a more severe hypertrophic phenotype (maxLVWT = +1.6 mm per PGS s.d., 95% CI = 0.61–2.63, *P* = 0.002; left ventricular mass = +13.8 g per PGS s.d., 95% CI = 1.5–26.2, *P* = 0.03) with no difference seen in the overall cohort of 440 cases (Supplementary Fig. 10 and Supplementary Table 13).

## Discussion

In this study, we generated a PGS for HCM and validated it across several independent populations, showing associations with categorical disease status, quantitative traits that define HCM and describe disease severity, and, most importantly, adverse cardiovascular events. We demonstrate broad potential clinical utility for PGS across a range of settings. Notably, PGS robustly stratifies penetrance in carriers of rare pathogenic variants in sarcomere-encoding genes and identifies those in the general population at the highest risk of developing HCM, associates with HCM risk in relatives of HCM probands and acts as a new risk marker for survival and adverse events in individuals with HCM.

Findings from this study emphasize the importance of the polygenic contribution to HCM disease risk, classically considered a Mendelian disease caused by rare variants in sarcomere-encoding genes^4,21^. Among individuals with HCM, the recognition of polygenic, rather than sarcomeric HCM, will be of diagnostic importance, with potential implications for clinical management, reproductive counseling and family screening^22^.

One of the key challenges in clinical practice remains understanding the variable penetrance and expressivity that characterize rare variants in HCM-causing sarcomeric genes^2,3^. In relatives of patients with HCM who have inherited a pathogenic variant, clinical screening and life-long surveillance from childhood are recommended, although many will not manifest until later life, if at all, and many of those who do manifest will follow a benign course without major adverse events. Increasingly, pathogenic variants are being identified in individuals with no personal or family history of HCM, as secondary findings through opportunistic screening alongside genetic testing for other indications^23^. We show that PGS has large and clinically meaningful effects (estimated to be approximately tenfold when comparing quintiles but larger still at more extremes of distribution) in carriers of rare HCM-causing variants, which we expect would translate to effective risk-adjusted strategies for HCM screening and surveillance. Furthermore, while current medical treatments are only indicated in individuals with established HCM, any future development of therapies in the prevention of cardiac hypertrophy in at-risk or genetically susceptible individuals could be of particular significance in the groups with the highest risk of disease penetrance. This is especially true given that both rare variants and PGS are measurable before clinical phenotypes of HCM develop.

In relatives of individuals with HCM, PGS risk modulates HCM penetrance in carriers of rare variants and stratifies HCM risk in sarcomeric gene-negative cases. Relatives with higher PGS were more likely to have HCM, adverse outcomes and increased wall thickness, with similar magnitudes of risk in relatives of both sarcomeric gene-positive and negative HCM. Importantly, relatives in the bottom PGS quintiles had low rates of lifetime adverse events. Directly quantifying PGS with genotyping arrays may guide ongoing surveillance strategies in close relatives of affected individuals, although further prospective work is required.

Within the general population, although PGS confers an increased risk of HCM at the extremes of distribution (OR = 15 for the highest centile compared with median), this risk was considerably lower than the risk arising from pathogenic rare variants (OR = 79). Our population estimates of PGS performance in the UKB are limited by recruitment targeting participants of middle age, with survival bias and incomplete ascertainment of cases likely to result in underestimation of the true effect of PGS in the general population. The applicability of routine and widespread use of PGS for disease screening remains uncertain^24^. As with any population screening approach, targeting PGS screening to individuals already at higher risk based on nongenetic factors can have a large impact on the numbers needed to test.

Among individuals with HCM, disease expressivity and prognosis are highly variable^4,21,25^. We demonstrate that PGS can stratify the risk of serious adverse events in individuals with HCM, including a roughly fourfold to sixfold difference in risk of death when comparing those with PGS in the highest and lowest quintiles. Despite the use of current clinical risk predictors of adverse outcomes^3^, many individuals do not benefit from interventions aimed at reducing this (for example, an implantable cardioverter-defibrillator^26^). The addition of PGS to existing clinical risk factors will be an important area for future research.

One of the main limitations of this and other PGS is that it has been derived from and extensively tested in European ancestry populations only. Despite this, we show that PGS stratifies ancestral risk, with the highest PGS found in African ancestry groups where the prevalence of unexplained left ventricular hypertrophy is known to be highest^27–30^. Within each non-European ancestry group in the UKB, performance is reduced compared to European ancestry performance, although it still associates with HCM-related cardiac traits in South Asian and East Asian populations. Furthermore, the addition of a small and individually underpowered ancestry-specific GWAS (East Asian) can improve the predictive performance of PGS. This lends hope that performing modestly sized ancestry-specific GWAS could be sufficient to generate PGS with comparable performance to European ancestry populations^16^.

In conclusion, this study identifies multiple clinical applications for a PGS in HCM, including in general population screening, stratification of rare variant carriers into higher and lower risk of penetrant HCM, and as a new risk predictor of adverse outcomes in individuals with HCM.

## Methods

### Ethics declaration

All patients gave written informed consent, and all studies were approved by the relevant regional research ethics committees and adhered to the principles set out in the Declaration of Helsinki. The UKB study was reviewed by the National Research Ethics Service (11/NW/0382 and 21/NW/0157). The 100,000 Genomes Project was reviewed by the National Research Ethics Service (14/EE/1112 and 13/EE/032). The RBH Biobank was reviewed and approved by the South Central—Hampshire B Research Ethics Committee (09/H0504/104+5 and 19/SC/0257). The Erasmus Medical Center was reviewed and approved by the Erasmus MC Medical Ethical Review Committee. All Singaporean participants recruited from the National Heart Center Singapore gave written informed consent and the study was approved by the Singhealth Centralised Institutional Review Board (2020/2353) and the Singhealth Biobank Research Scientific Advisory Executive Committee (SBRSA 2019/001v1).

### GWAS and multitrait analysis

The base data for the HCM PGS are from the largest HCM GWAS, consisting of 5,900 cases and 68,359 unrelated controls from seven cohorts (100,000 Genomes Project (471 cases, 2,355 controls), BioResource Rare Diseases (239 cases, 7,203 controls), HCM Registry (2,431 cases, 40,283 controls) and clinical cohorts from Canada (1,035 cases, 13,889 controls), Italy (277 cases, 1,293 controls), the Netherlands (999 cases, 2,117 controls) and the UK (448 cases, 1,219 controls)^10^. HCM was defined as primary left ventricular hypertrophy in the absence of secondary causes (uncontrolled hypertension, aortic valve disease, infiltrative cardiomyopathic processes and cases arising from complex syndromes), using a combination of clinical, imaging and ICD-9 and ICD-10 definitions. Detailed information on cohorts included in the GWAS is provided in the original publication^10^. Cases and controls included in the HCM GWAS were of European ancestry. The heritability of HCM attributable to common genetic variants was 0.25, assessed using genome-based restricted maximum likelihood^31^. Leveraging the increased power generated from jointly analyzing genetically correlated traits using the MTAG method^32^, MTAG of HCM was performed using mtag^32^ with three genetically correlated quantitative left ventricular traits derived from CMR imaging in 36,083 participants in the UKB (left ventricular concentricity, LVESV and left ventricular circumferential strain)^10^. Traits were selected based on the hierarchical clustering of ten CMR traits and genetic correlation with HCM. Additional details of left ventricular trait GWAS and trait selection for MTAG are reported in the Supplementary Note.

### PGS derivation and evaluation

Individual SNP weighted scores were generated from the primary discovery GWAS and MTAG. The base GWAS and MTAG summary statistics were filtered to exclude rare and uncommon variants (minor allele frequency (MAF) < 1%), and ambiguous SNPs that were not resolvable by strand flipping. A locus on chromosome 11 surrounding *MYBPC3* was found to be associated with HCM in only sarcomere-positive HCM, specifically in one cohort (Netherlands), and was determined to represent a founder effect^10^. Variants with *P* < 1 × 10^−^^5^ on chromosome 11 from 30,000,000 to 80,000,000 (GRCh37) were excluded from PGS calculation.

We calculated HCM PGS for unrelated (third degree or closer) white British participants in the UKB (application, 47602), using variants that passed genotyping QC (MAF > 1%, genotyping rate > 0.99, HWE *P* > 1 × 10^−^^6^). Variants overlapping the base, target and linkage disequilibrium reference set (1000 Genomes Project Phase 3 European ancestry) were included. The individual SNP scores were generated using PRS-CS v1.0, a package that uses a Bayesian framework to model linkage disequilibrium using an external linkage disequilibrium reference set and a continuous shrinkage prior on SNP effect sizes^33^. The phi constant was automatically selected by an unsupervised approach (PRS-CS auto). Whole-genome PGS scores for all included UKB individuals and testing cohorts were calculated using the ‘score’ function in PLINK v1.9 (ref. ^34^).

PGS was applied and tested within a range of cohorts and clinical settings. Given that a key factor in the predictive power of PGS is the power of the base GWAS^35^, we first compared the performance of PGS generated using GWAS (PGS_GWAS_ = 376,730 SNP predictors) and MTAG^10^ (PGS_MTAG_ = 374,113 SNP predictors) summary statistics in 343,182 unrelated white British ancestry participants in the UKB. Predictive performance of PGS was assessed by comparing Nagelkere’s *R*^2^, area under the receiver operating characteristic (AUROC) and association with HCM (OR per PGS s.d.).

Inclusion of participants in both the testing and GWAS datasets results in substantial inflation of PGS performance^36^. To prevent this, where case–control PGS testing was performed in a cohort that was included in the main GWAS (for example, GeL), PGS was generated using a leave-one-study-out GWAS and MTAG that did not include the cohort. All other methods for PGS generation remained the same. In situations where only cases are included in the assessment of PGS, the overall MTAG results were used.

### Cohorts

#### UKB

UKB is a population-based cohort study from half a million UK participants, with detailed clinical, imaging and genetic data. Participants from UKB that were included in testing were unrelated (third degree or closer) and of white British ancestry. HCM cases were identified from self-report clinical data (hospital admissions and death registry), and CMR imaging (maxLVWT > 15 mm). Time to clinical event was identified from UKB first occurrences data, operation dates, and death dates. Participants in the imaging substudy were randomly invited from the overall cohort. Each underwent CMR at 1.5-T. Segmentation of the cine images was performed by using a deep learning neural network algorithm and has previously been reported^6^.

#### 100,000 Genomes Project

The 100,000 Genomes Project is a national UK program that recruited probands with rare diseases and cancer from clinical centers, together with family members, and performed germline and somatic (for a subset of participants with cancer) WGS^18,37^. In total, 683 HCM cases were identified from Human Phenotype Ontology terms at the time of study recruitment, and ICD-9 and ICD-10 codes from preceding and subsequent clinical episodes.

#### EMC cohort

To evaluate the role of PGS in modulating penetrance of sarcomeric variants in relatives of HCM cases, we used a subset of 214 relatives of 184 HCM probands from an ongoing HCM registry at the EMC^38,39^. All individuals were carriers of pathogenic sarcomeric variants, with the exclusion of homozygous carriers or those carrying multiple pathogenic or likely pathogenic variants.

#### Royal Brompton and Harefield Hospitals cohort

A total of 440 unrelated white British HCM cases from RBH^40^ were used to assess the effect of PGS on CMR imaging traits. Data from the clinical CMR scan taken at or before study recruitment were used, and where sequential CMR scans were available, follow-up imaging data was recorded to identify changes in imaging traits.

### Statistical analysis

In the UKB, PGS model performance was assessed using Nagelkerke’s *R*^2^, adjusting the null model for age, age^2^, sex and first ten principal components. The predictive AUROC was determined using a randomly subsetted training (70%) and validation (30%) cohort using R-package pROC (v1.18.0)^41^. For association between PGS and HCM status in UKB and GeL, logistic regression was performed adjusting for age, age^2^, sex and first ten principal components. In EMC, this was assessed using Wald logistic mixed-effects model using GMMAT (v1.3.2) adjusting for fixed-effects of sex, age, age^2^ and first four principal components and incorporating a genetic relatedness matrix estimated using GCTA (v1.92.2beta)^42^ as a random effect. For quantitative imaging traits in UKB and RBH, PGS association was evaluated using linear regression adjusting for age, age^2^, sex, first ten principal components, systolic blood pressure and body surface area, and differences between means in stratified groups were performed with ANCOVA testing adjusted for age, age^2^, sex, body surface area, systolic blood pressure and first ten principal components. In EMC, the association of PGS with maxLVWT was assessed using a linear mixed-effects model using coxme (v2.2-17)^43^, adjusting for sex, age at imaging, age at imaging^2^, imaging modality (CMR versus transthoracic echocardiogram), first four principal components and the genetic relatedness matrix. Time-to-event data in UKB was evaluated using the Cox proportional hazards model, adjusting for age, age^2^, sex and first ten principal components using survival (v2.44-1.1). Hazards assumption for proportionality was assessed, and for outcomes that did not include death, a competing risk analysis was performed. In EMC, the association between PGS and clinical events was assessed using a Cox proportional hazards mixed-effects model using R-package coxme (v2.2-17)^43^ adjusted for sex, first four principal components, genetic relatedness matrix and presence of MYH7 rare variant genotype status. Time-to-event analysis was performed using survival (v3.5-7) and survival curves were created using survminer (v0.4.9). Although clinical data were complete for most individuals in all cohorts, where missing data was present, individuals were excluded from analysis. All statistical analysis was performed in R. For multi-ancestry analysis, ancestry as categorical variable was included in the regression model.

### Rare variant status

The pathogenicity of rare variants in eight definitive HCM-causing genes^17^ (*MYBPC3*, *MYH7*, *TNNT2*, *TNNI3*, *TPM1*, *ACTC1*, *MYL3* and *MYL2*) was determined using broadly similar approaches across cohorts in line with ACMG guidelines^44^ (Supplementary Note). Individuals without pathogenic or likely pathogenic variants were identified as gene-negative individuals.

### Outcomes

For a diagnosis of HCM in the UKB, HCM cases were identified from self-reporting, ICD-9 and ICD-10 codes from hospital encounters and the national death register, and CMR imaging (maxLVWT > 15 mm), in the absence of aortic stenosis (Supplementary Note). For the analysis of imaging traits in HCM cases, we further refined the diagnosis by restricting only to individuals with a maxLVWT of at least 13 mm. PGS association with a range of HCM-relevant cardiac imaging traits associated with cardiac structure (maxLVWT, LVEDV, LVESV, left atrium volume and fractal dimensions) and function (LVEF, and strain measurements) was tested. Longitudinal risk of time to HCM diagnosis and for major adverse cardiovascular events was assessed. Clinical and operative outcomes were selected based on their relevance to HCM, incorporating self-reported diagnoses, hospital admission events, primary care records and death records (Supplementary Note). Diagnosis of HCM in additional cohorts (EMC, GeL and RBH) and clinical outcomes in EMC are reported in Supplementary Note.

### PGS generation and testing in diverse ancestry groups

PGS generated using European ancestry GWAS have weaker performance when tested in more diverse ancestry populations^12–15^. We first aimed to evaluate PGS performance in participants of Afro-Caribbean (*n* = 661), East Asian (*n* = 504) and South Asian (*n* = 489) ancestry groups in UKB by applying ancestry-specific 1000 Genomes Project linkage disequilibrium reference sets to the European ancestry GWAS and MTAG when generating PGS. Ancestries of UKB participants were determined based on self-reported ancestry, followed by visualization of principal component plots and manual selection of principal component thresholds. Given that PGS are not comparable between differing ancestries due to underlying differing genetic architecture, analyses using PGS as a continuous variable were restricted within single ancestry groups. For analysis stratifying by quantiles, quantile stratification was first performed within each ancestry before being combined with other ancestries.

PRS-CSx v1.0 was used to extend the Bayesian polygenic modeling and prediction methods of PRS-CS by combining GWAS summary statistics from multiple ancestry groups and has been shown to improve cross-ancestry prediction^16^. We aimed to evaluate the performance of PGS generated using this approach for the prediction of HCM-associated CMR traits in East Asian ancestry participants in the UKB by combining the European ancestry GWAS with a small East Asian ancestry GWAS (Singapore cohort).

### Singapore HCM GWAS

GWAS was performed on 184 cases and 776 controls of East Asian ancestry. Genotyping was performed using Infinium OmniExpress-24 kit (Illumina). Imputation was performed on the Michigan Imputation Server^45^ using Minimac4 (v1.5.7) and East Asian reference genomes (1000 Genomes Phase 3 (v5)^46^, 1000 Genomes Phase 1 (v3)^46^ and Genome Asia Pilot^47^). Postimputation QC was performed at variant (HWE *P* > 1 × 10^−^^7^, genotyping > 0.95, information score > 0.5 and MAF > 1%) level. East Asian ancestry individuals were identified using principal component analysis and one of a pair of second degree or closer relatives was retained. GWAS was tested using SNPTEST (v2.5.6)^48^ adjusting for age, sex and first ten principal components.

### PheWAS and MR

PheWAS was performed in the UKB to investigate the pleiotropic effects of the HCM PGS. ICD-9 and ICD-10 codes from death records and hospital admission episodes were translated to Phecodes (Phecode Map 1.2)^49,50^. For phenotypes with at least 20 cases, PGS-phenotype association was tested using logistic regression adjusted for age, age^2^, sex and first ten principal components as covariates. Significance threshold was adjusted for the total number of phenotypes tested (*P* < 2.72 × 10^−^^5^), and data were presented with Manhattan plots grouping by body system. PheWAS was performed using the PheWAS (v0.99.5-5)^51^ in R (v4.0.3).

To further evaluate the directionality of effect for select significant PheWAS associations (hypertension, dyslipidemia, type 2 diabetes), two-sample bidirectional MR was performed for relevant quantitative traits (systolic and diastolic blood pressure, hypercholesterolemia, glycated hemoglobin and body mass index). To maximize MR power, the exposure trait GWAS with the largest number of significant SNPs after harmonization from the IEU GWAS database was used as the instrument^52–55^ (Supplementary Table 8). To further evaluate associations with atrial fibrillation and heart failure, post hoc MR was performed using summary statistics of the two largest available published GWAS^56,57^. For all MR analyses, instruments were harmonized with the HCM MTAG after linkage disequilibrium pruning. Two-sample MR using the IVW method was performed using the TwoSampleMR (v0.6.4)^58,59^ and MRInstruments (v0.3.2) in R (v4.0.3).

### Reporting summary

Further information on research design is available in the Nature Portfolio Reporting Summary linked to this article.

## Online content

Any methods, additional references, Nature Portfolio reporting summaries, source data, extended data, supplementary information, acknowledgements, peer review information; details of author contributions and competing interests; and statements of data and code availability are available at 10.1038/s41588-025-02094-5.

## Supplementary information

Supplementary Information Supplementary Figs. 1–10 and Supplementary Note.

Reporting Summary

Supplementary Tables Supplementary Tables 1–13.

## Data Availability

Data from UKB can be requested from the UKB Access Management System (https://www.ukbiobank.ac.uk/enable-your-research/apply-for-access). Data from the 100,000 Genomes Project can be accessed following the application to join the Genomics England Clinical Interpretation Partnership (https://www.genomicsengland.co.uk/research/academic/join-research-network). The PGS are available for download from the Polygenic Score Catalog (https://www.pgscatalog.org) under accessions PGS004910 and PGS004911. GWAS and MTAG results^10^ used to generate PGS are available for download from the GWAS Catalog (https://www.gwascatalog.org) under accessions GCST90432127 and GCST904321230.

## Code availability

Custom analysis code is available from Zenodo (10.5281/zenodo.11204463)^60^.
