# Supplementary material for: Evaluation of polygenic scores for hypertrophic cardiomyopathy in the general population and across clinical settings
Source: Nat Genet. 2025 Feb 18;57(3):563–71. doi: 10.1038/s41588-025-02094-5 (PMC11906360; doi:10.1038/s41588-025-02094-5)
Supplement: Supplementary file 1 — Supplementary Figs. 1–10 and Supplementary Note. [file 41588_2025_2094_MOESM1_ESM.pdf]

nature genetics

Supplementary information

https://doi.org/10.1038/s41588-025-02094-5

Evaluation of polygenic scores for hypertrophic cardiomyopathy in the general population and across clinical settings

In the format provided by the authors and unedited

Supplementary Figures

Supplementary Figure 1: PGS and cohort overview 2
Supplementary Figure 2: PGS in the general population 3
Supplementary Figure 3: HCM PGS and cardiac imaging traits in the general population 5
Supplementary Figure 4: Mendelian randomization of pheWAS significant associations 7
Supplementary Figure 5: Mendelian randomization of HCM as exposure on heart failure and atrial fibrillation 9
Supplementary Figure 6: PGS in non-European ancestry groups 11
Supplementary Figure 7: Singapore East Asian ancestry HCM case-control GWAS 13
Supplementary Figure 8: PGS in sarcomere-positive carriers 14
Supplementary Figure 9: PGS in SARC-PLP carrier relatives of HCM cases 16
Supplementary Figure 10: PGS predicts adverse events and disease severity in HCM cases 18
Supplementary Figure References 20

Supplementary Note

LV trait GWAS and multi trait analysis of GWAS 21
HCM definition across cohorts 23
Clinical outcomes in UK Biobank 24
Erasmus Medical Centre cohort methods 25
Variant pathogenicity 26
HCM GWAS collaborators 27
Supplementary Note references 28

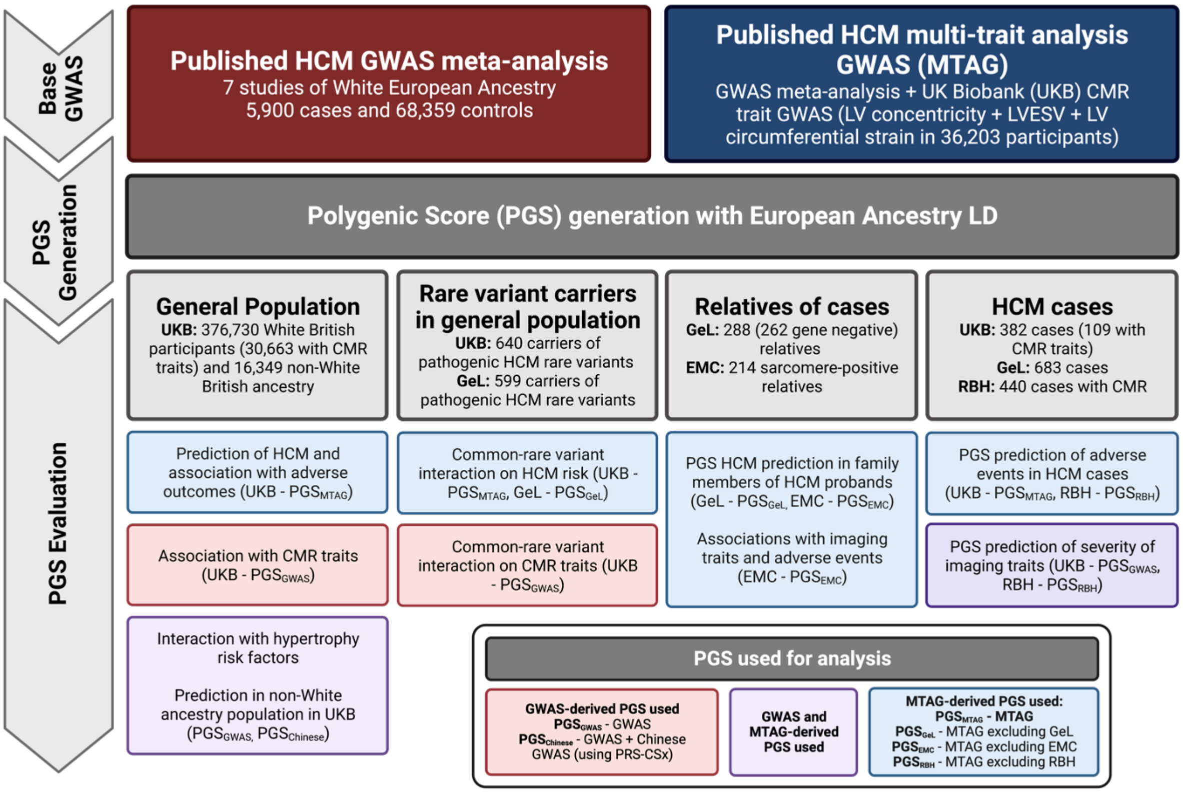

Supplementary Figure 1: PGS and cohort overview. Bayesian genome-wide PGS were generated from a published European-ancestry hypertrophic cardiomyopathy (HCM) GWAS meta-analysis of seven case-control studies (comprising 5,900 cases and 68,359 controls; PGS_{GWAS}), and multi-trait analysis of GWAS (analysing HCM with three genetically-correlated quantitative traits measured using cardiac MRI [CMR] in 36,203 UKB participants: LV concentricity, LV end systolic volume and LV circumferential strain; PGS_{MTAG}). In order to minimise inflation due to overlap of samples in the base GWAS and cohort in which the PGS is being evaluated, leave-one-study-out GWAS meta-analyses were performed to generate base GWAS without any sample overlap. For example, when PGS was evaluated in GeL, first a GWAS meta-analysis excluding the GeL cohort was performed, which was then used to generate PGS that was tested in GeL. Similarly, for association of CMR traits in the UKB, given that the MTAG used GWAS summary statistics of imaging traits performed in the UKB, PGS derived from GWAS meta-analysis (PGS_{GWAS}) was used rather than PGS derived from MTAG. All PGS performed similarly well in their associations with population risks of HCM in the UKB (Table S1). UKB – UK Biobank, GeL – 100,000 Genomes Project; EMC – Erasmus Medical Centre, Netherlands; RBH – Royal Brompton Hospital, UK. LV – left ventricle/ventricular, LVESV – LV end-systolic volume.

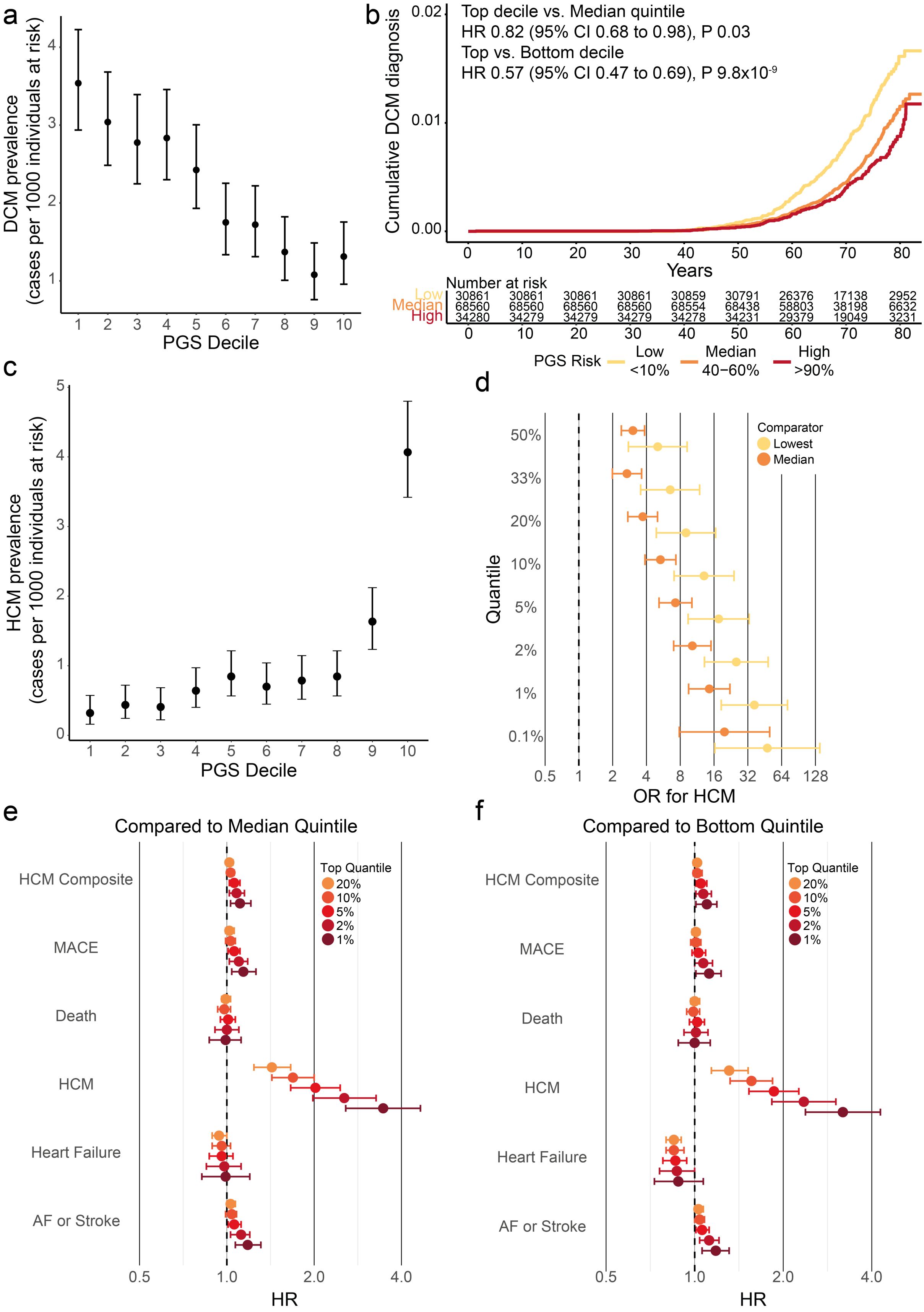

Supplementary Figure 2: Polygenic scores in 343,182 participants from the general

population. (a) Prevalence of dilated cardiomyopathy (DCM) in the UKB in each HCM-PGS decile. DCM defined using self-reported and ICD-10 codes (I42.0, I42.6, I42.7, and O90.3), in the absence of coronary artery disease and congenital heart disease. Data presented as prevalence with a binomial 95% confidence interval (CI) for each PGS decile. **(b)** Time to DCM diagnosis in top decile, median quintile and bottom deciles in UKB. Hazards ratio calculated using Cox proportional hazards model, adjusted for age, age^2, sex, and first ten genetic PCs, with two-sided P-value. **(c)** Prevalence of HCM in the UKB in each PGS decile. Data presented as prevalence with a binomial 95% confidence interval for each decile. **(d)** OR for HCM comparing a range of top quantiles with median 20% and lowest quantiles, with 95% CI. **(e,f)** Hazards ratio for adverse cardiovascular events when comparing the top quantile with the median **(e)** and bottom **(f)** quintiles. Data presented as hazard ratios with 95% CI. Full results for the top centile, along with individual components of composite outcomes is reported in **Supplementary Table 5**. HCM composite consists of death, heart failure, AF, stroke, cardiac arrest, septal reduction therapy (surgical myectomy or alcohol septal ablation), ICD implantation, LVAD implantation, or cardiac transplantation. MACE consists of HCM diagnosis, heart failure, AF, stroke or cardiac arrest.

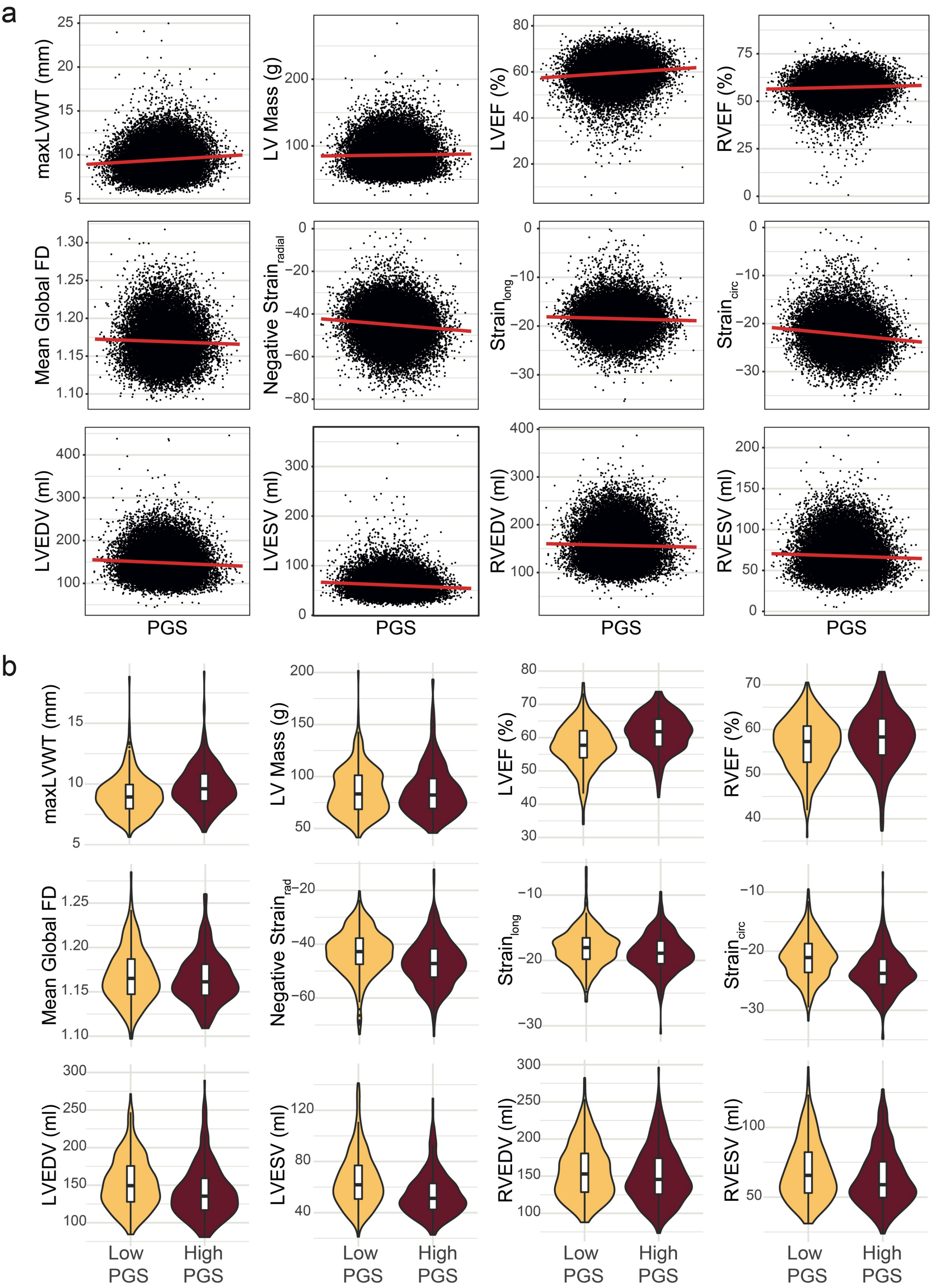

Supplementary Figure 3: HCM PGS and cardiac imaging traits in the 30,663 individuals in the general population. (A) PGS_{GWAS} associations with machine-learning derived quantitative CMR traits in 30,663 unrelated participants in the UKB. Univariate regression line of PGS and trait (red line). Linear regression adjusting for age, age^2, sex, body surface area,

systolic blood pressure at time of scan, and top ten genetic PCs. Two-sided FDR-adjusted P-values presented. (B) Violin plot of quantitative CMR traits in top (N=305) and bottom (N=304) PGS centiles. Box plots indicate mean and standard deviation. Changes in quantitative CMR traits per SD and comparing top centile with median and bottom quintiles is presented in Tables S6 and S7. LV – left ventricle/ventricular; maxLVWT – maximum LV wall thickness; LVEF – LV ejection fraction; RV – right ventricle/ventricular; RVEF – RV ejection fraction; FD – fractal dimension; $strain_{radial}$ – mean global LV radial strain; $strain_{long}$ – mean global LV longitudinal strain; $strain_{circ}$ – mean global LV circumferential strain; LVEDV – LV end-diastolic volume; LVESV – LV end-systolic volume; RVEDV – RV end-diastolic volume; RVESV – RV end-systolic volume.

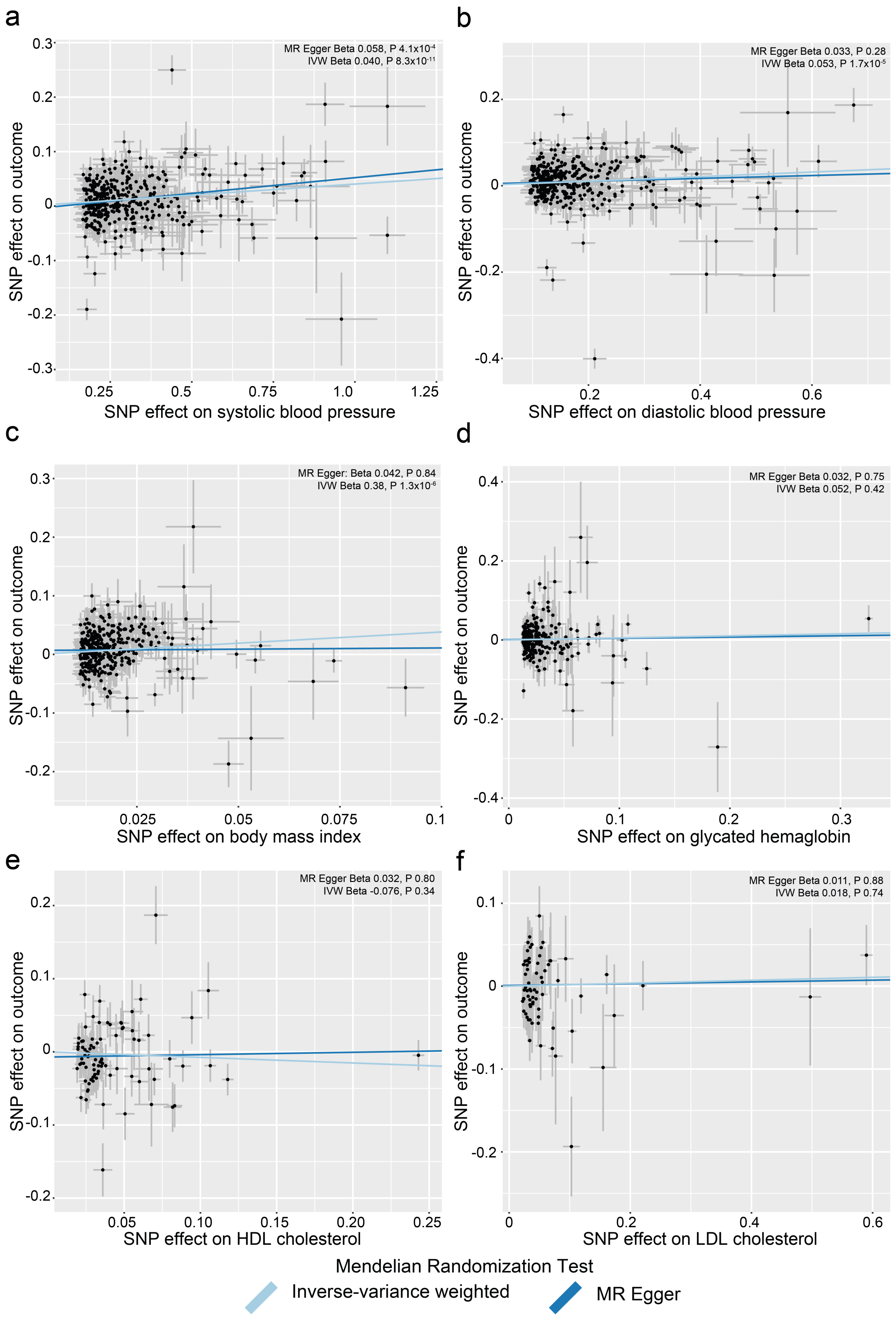
a
MR Egger Beta 0.058, P 4.1x10-4
IVW Beta 0.040, P 8.3x10-11
SNP effect on outcome
SNP effect on systolic blood pressure
b
MR Egger Beta 0.033, P 0.28
IVW Beta 0.053, P 1.7x10-5
SNP effect on outcome
SNP effect on diastolic blood pressure
c
MR Egger: Beta 0.042, P 0.84
IVW Beta 0.38, P 1.3x10-6
SNP effect on outcome
SNP effect on body mass index
d
MR Egger Beta 0.032, P 0.75
IVW Beta 0.052, P 0.42
SNP effect on outcome
SNP effect on glycated hemaglobin
e
MR Egger Beta 0.032, P 0.80
IVW Beta -0.076, P 0.34
SNP effect on outcome
SNP effect on HDL cholesterol
f
MR Egger Beta 0.011, P 0.88
IVW Beta 0.018, P 0.74
SNP effect on outcome
SNP effect on LDL cholesterol
Mendelian Randomization Test
Inverse-variance weighted
MR Egger

Supplementary Figure 4: Mendelian randomization of pheWAS significant associations. Relationship of SNP effects on the exposure trait: systolic **(a)** and diastolic blood pressure **(b)**, body mass index **(c)**, glycated haemoglobin **(d)**, and HDL **(e)** and HDL cholesterol **(f)**; with HCM for 2 Mendelian randomization test methods (inverse variance weighted [IVW], and MR Egger). Genetic instruments were extracted from large-scale published GWAS (blood pressure traits from GWAS of 757,601 individuals[1], glycemic traits from 281,416 individuals[2], lipid traits from GWAS of 188,577 individuals[3], and BMI from 461,460 individuals[4]). Error bars are 95% confidence intervals for SNP effects. Slope of the line is the beta of the MR test method. Two-sided unadjusted P-values are reported.

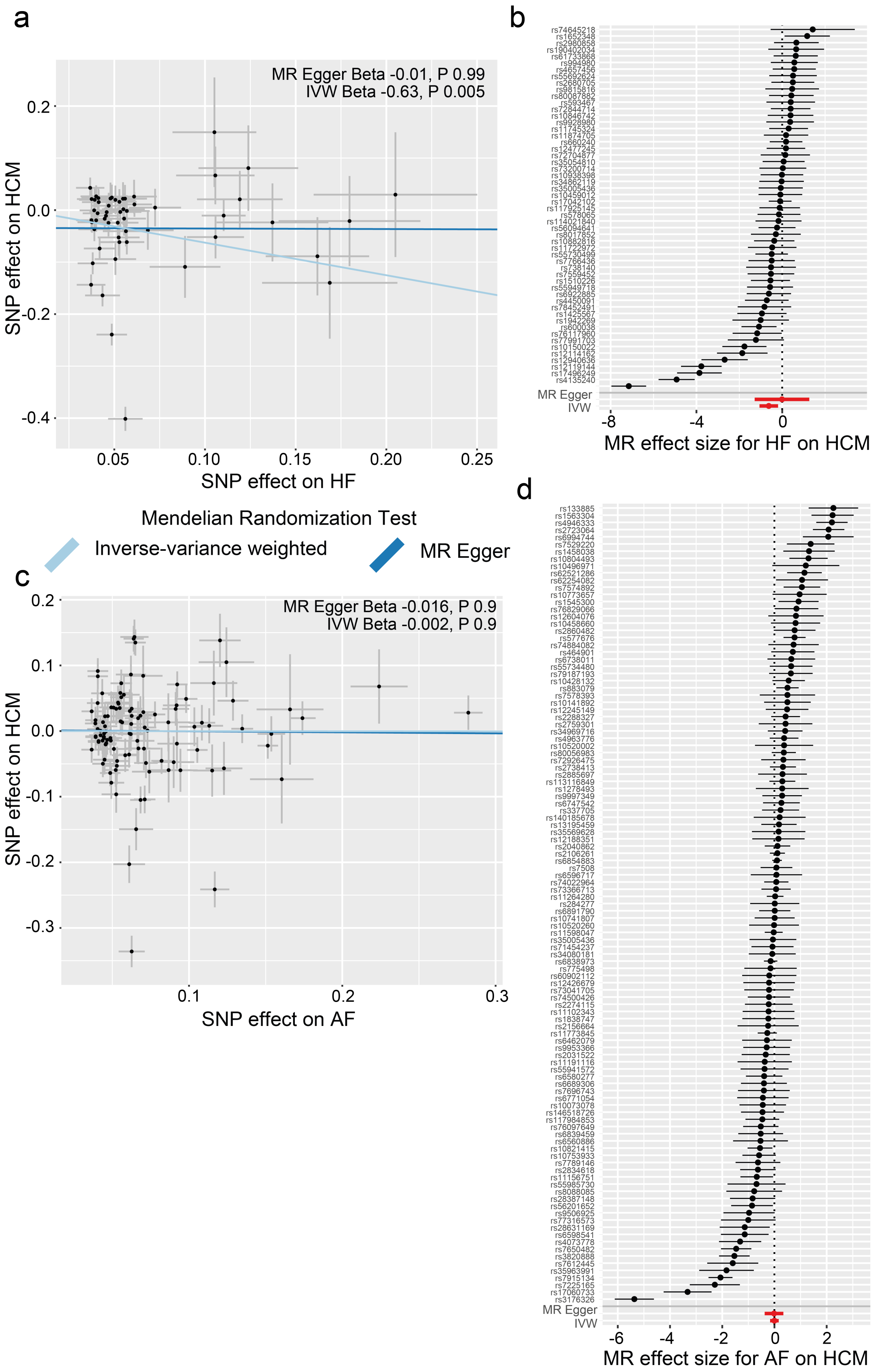
a
MR Egger Beta -0.01, P 0.99
IVW Beta -0.63, P 0.005
SNP effect on HCM
SNP effect on HF
0.2
0.0
-0.2
-0.4
0.05
0.10
0.15
0.20
0.25
b
MR Egger
IVW
-8
-4
0
MR effect size for HF on HCM
Mendelian Randomization Test
Inverse-variance weighted
MR Egger
c
MR Egger Beta -0.016, P 0.9
IVW Beta -0.002, P 0.9
SNP effect on HCM
SNP effect on AF
0.2
0.1
0.0
-0.1
-0.2
-0.3
0.1
0.2
0.3
d
MR Egger
IVW
-6
-4
-2
0
2
MR effect size for AF on HCM

Supplementary Figure 5: Mendelian randomization of HCM as exposure on heart failure and atrial fibrillation. Relationship of SNP effects of HCM as the exposure on heart failure[5] **(a,b)** and atrial fibrillation[6] **(c,d)**, using two Mendelian randomization test methods (inverse variance weighted [IVW], and MR Egger). Effect-effect plots **(a,c)** shown for included instruments. Error bars are 95% confidence intervals for SNP effects. Slope of the line is the beta of the MR test method. Two-sided unadjusted P-values are reported. Forest plots **(b,d)** shown to compare the MR estimates against individual SNP results. Effect size and 95% confidence intervals reported. Genetic instruments were extracted from large-scale published GWAS of heart failure[5] (47,309 cases and 930,014 controls) and atrial fibrillation[6] (60,620 cases and 970,216 controls).

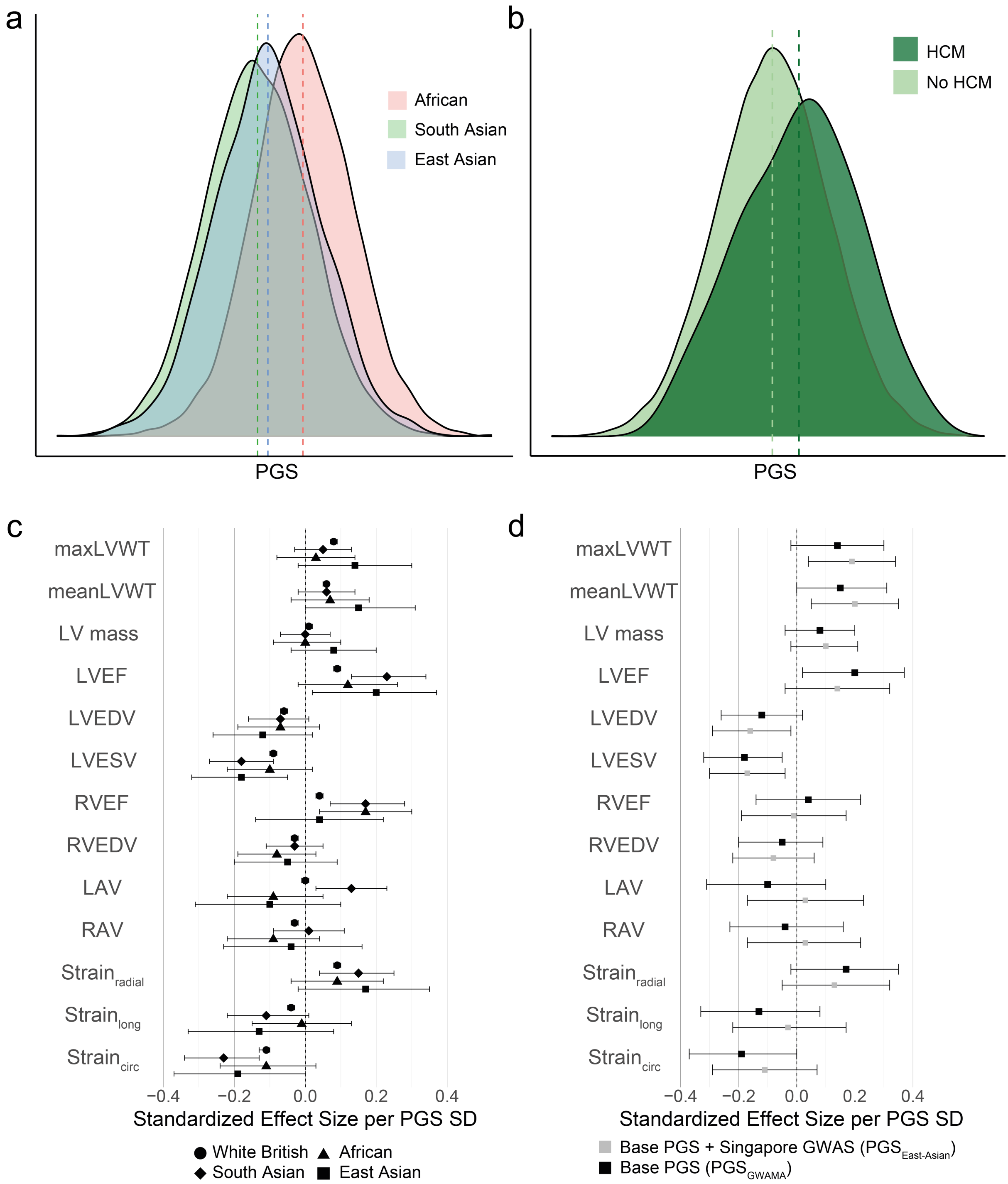

Supplementary Figure 6: PGS in non-European ancestry groups. (a) PGS distribution across ancestries in South Asian (9 HCM cases in 7,542 participants, 0.1%), Afro-Caribbean (27 cases in 7,346, 0.4%) and East Asian (2 cases in 1,457, 0.1%) participants in the UKB, and in non-European British case and controls. **(b)** Pooled PGS distribution in non-European ancestry individuals in UKB, stratified by case-control status. **(c)** Standardised effect size per PGS SD on CMR traits in non-European ancestry groups (Afro-Caribbean: N=7,346; South Asian: N= 7,538; East Asian: N=1,457). Full results in Table S9. **(d)** Standardised effect size per PGS SD on CMR traits in 1,457 East Asian ancestry individuals in UKB using standard PGS (PGS_{GWAS}) and PGS generated with the addition of a GWAS of 184 cases and 776 controls of East Asian ancestry, using PGS-CSx ($PGS_{East\text{-}Asian}$). Full results in Table S10. For

(c,d), effect estimates were generated using linear regression adjusting for age, age^2, sex, systolic blood pressure, body surface area, and top ten genetic PCs, with unadjusted two-sided P value. Data are presented as effect estimates with 95% confidence intervals.

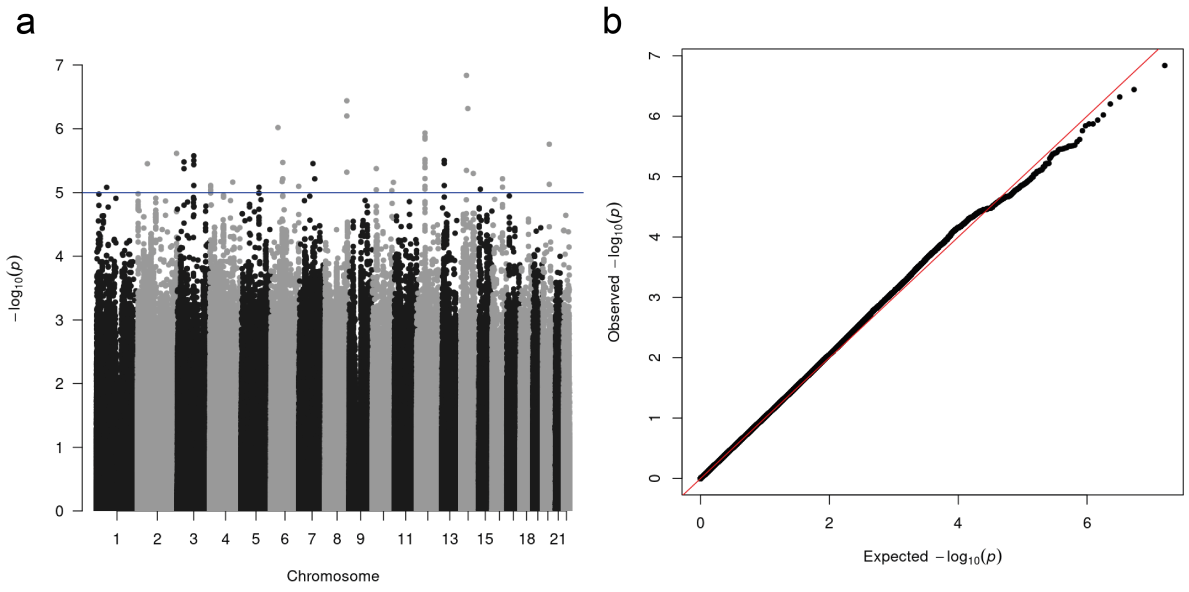

Supplementary Figure 7: Singapore East Asian ancestry HCM case-control GWAS. (a) Manhattan and **(b)** QQ plot of Singapore HCM case-control GWAS consisting of 174 HCM cases and 776 controls, all of East Asian ancestry (lambda GC 1.03). GWAS was tested using an additive model, adjusting for age, sex, and first ten principal components. P-values are two-sided and unadjusted. Blue line indicates suggestive significance threshold ($P<1x10^{-5}$). No SNPs reached genome-wide significance ($P<5x10^{-8}$).

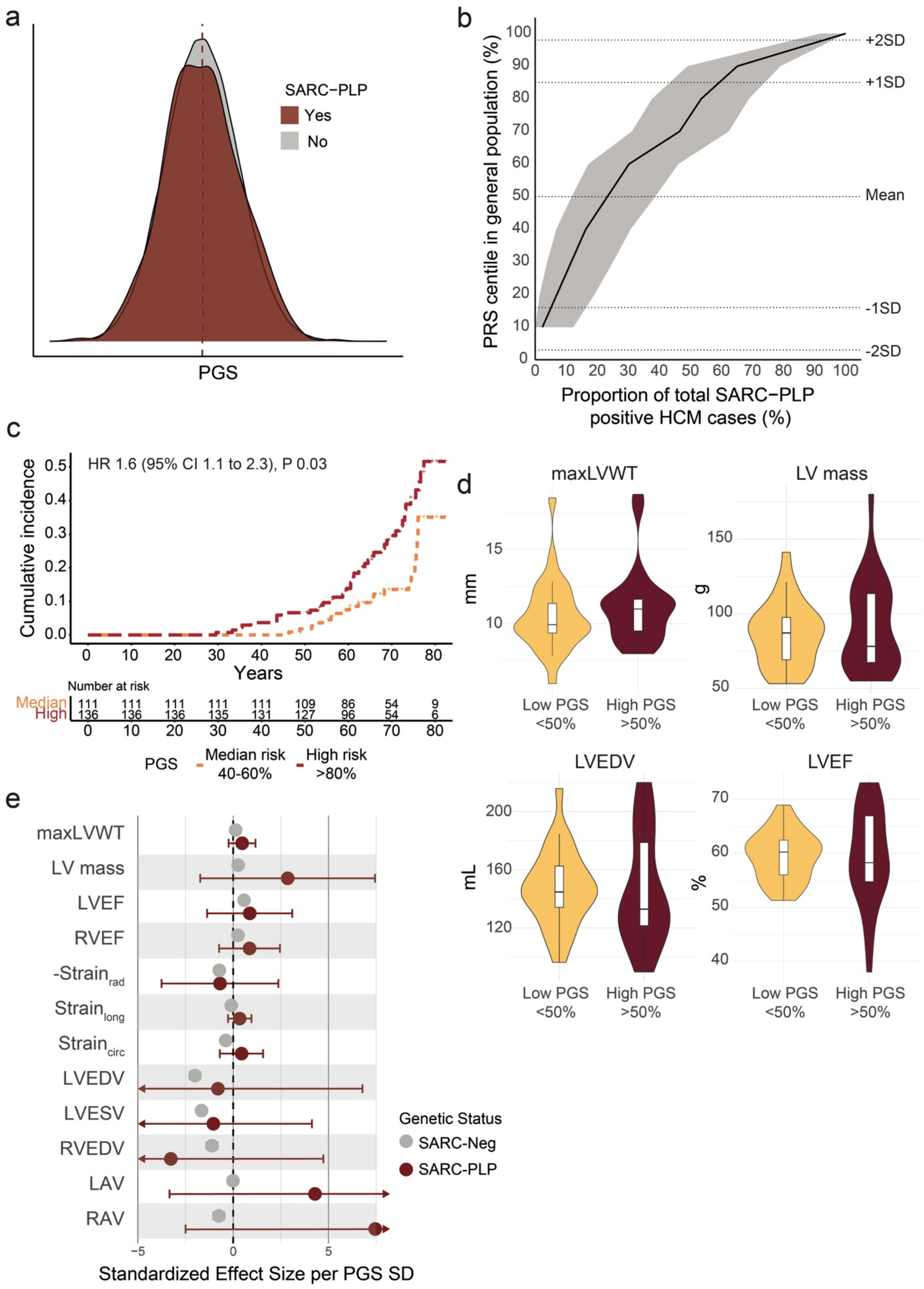

Supplementary Figure 8: PGS in sarcomere-positive carriers. (a) PGS distribution in 318,945 whole-exome sequenced UKB participants with (n=640) and without (n=318,305) pathogenic HCM-causing variants shows no difference between groups, suggesting again any

marked selection or suvival bias. **(b)** Cumulative curve of gene positive HCM cases across PGS centiles. Dashed lines represent population mean, ±1 SD and ±2 SD. **(c)** Time to HCM composite outcome (comprising death, cardiac arrest, atrial fibrillation, stroke, HCM, heart failure, ICD implantation, septal reduction therapy, LVAD implantation and cardiac transplantation) in top, median and bottom quintiles. Hazards ratio calculated using Cox proportional hazards model, adjusted for age, age^2, sex, and first ten genetic PCs, with two-sided P value. **(d)** Violin plot of quantitative CMR traits in individuals with a PGS above (N=26) and below (N=27) the median. Box plot indicating median and interquartile range, whiskers denoting 1.5-times the interquartile range, and the edges of violin plot indicating minimum and maximum values. **(e)** Standardized effect size of PGS SD on quantitative CMR traits known to be affected in HCM, in those with (N=53) and without (N=29,002) SARC-PLP variants highlighting directionally concordant trends in both groups. Data presented as standardized effect size for each SD increase in HCM PGS from a linear regression model with 95% confidence interval.

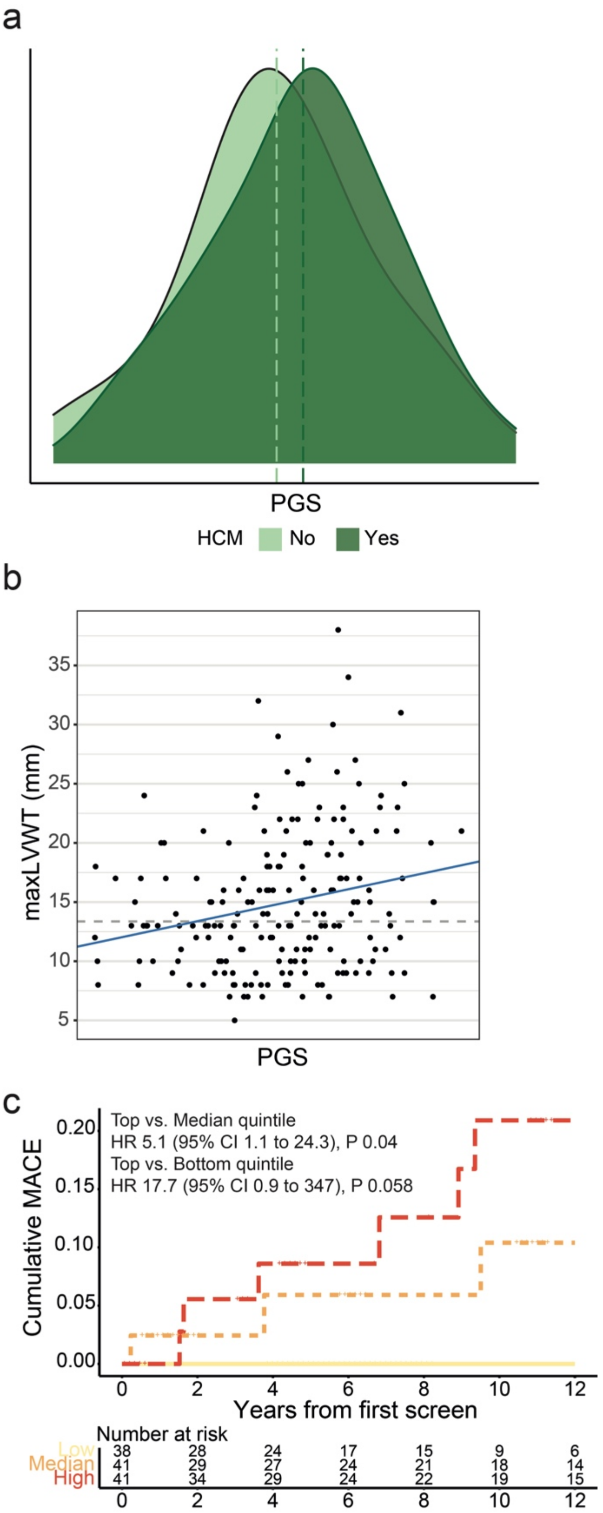

Supplementary Figure 9: PGS in SARC-PLP carrier relatives of HCM cases. (a) PGS distribution in 214 relatives of HCM cases in the Erasmus cohort, stratified by HCM status. **(b)** Scatter plot of maxLVWT against PGS_{EMC} among 194 sarcomere-positive relatives of HCM index patients from Erasmus cohort. Univariate linear regression line (blue line) and common diagnostic cutoff for HCM in relatives of cases (13 mm, grey dotted line). **(c)** Cumulative major

adverse cardiovascular events (MACE) after initial screening in sarcomere-positive relatives of HCM probands stratified by PGS_{EMC} in the top, middle and bottom quintiles. MACE was defined as a composite of septal reduction therapy, cardiac transplantation, aborted cardiac arrest, appropriate defibrillator shock, or sudden cardiac death. Hazards ratio calculated using Cox proportional hazards model, adjusted for sex, first four genetic PCs, and genetic relatedness matrix, with two-sided P value.

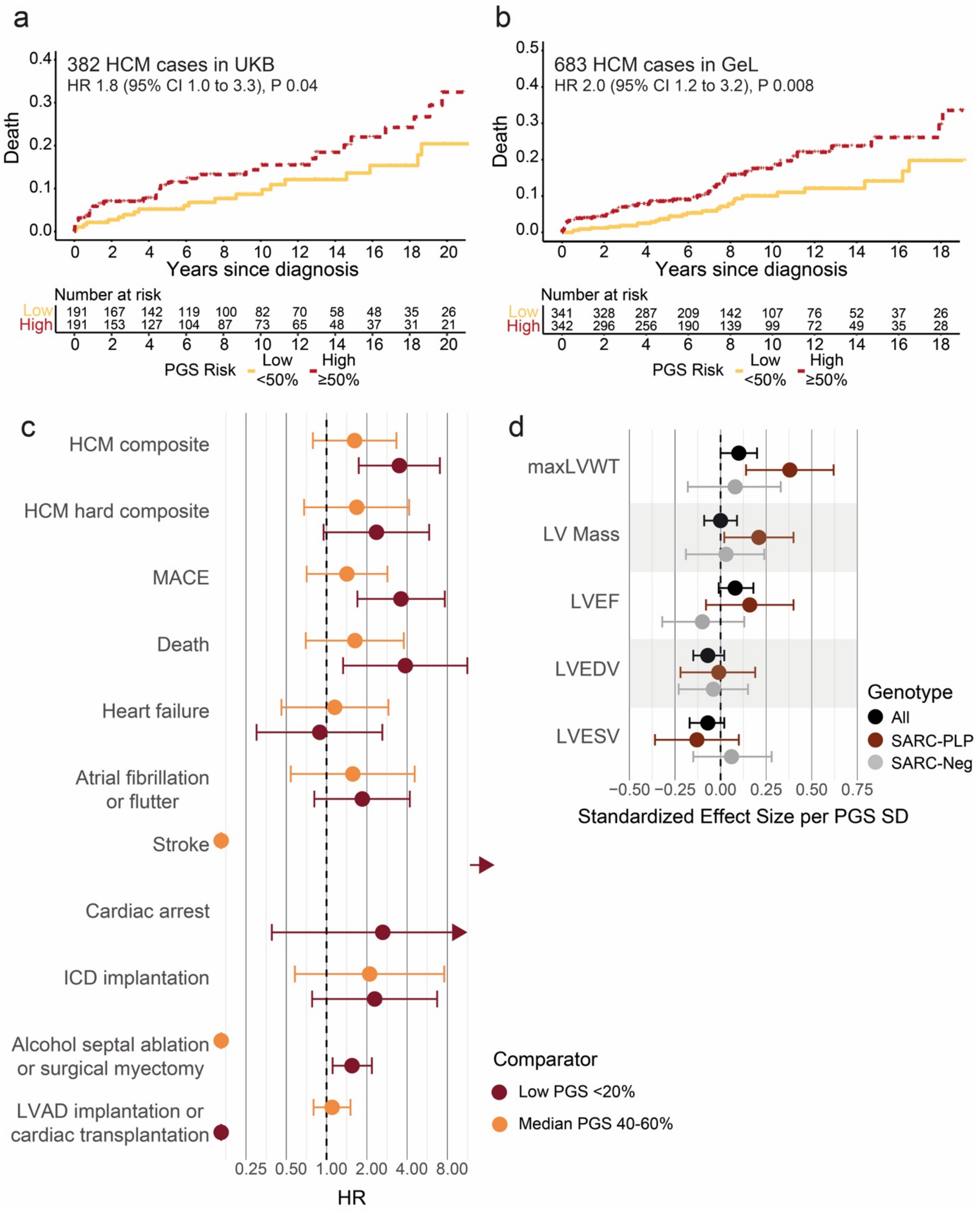

Supplementary Figure 10: PGS predicts adverse events and disease severity in HCM cases. Cumulative hazard curves for all-cause mortality in UKB (n=382) **(a)** and GeL (n=683) **(b)** after HCM diagnosis, stratified by median PGS. Hazards ratio calculated using Cox proportional hazards model, adjusted for age, age^2, sex, and first ten genetic PCs, with two-sided P value. **(c)** Risk of adverse events in HCM cases, comparing top with median and bottom PGS quintiles in 382 HCM cases in the UKB. Data presented as hazard ratios with 95% confidence intervals. Full results in **Supplementary Table 12**. HCM composite adverse outcome consists of death, heart failure, atrial fibrillation, stroke, cardiac arrest, septal reduction therapy (myectomy or alcohol septal ablation), ICD implantation, LVAD implantation

or cardiac transplantation. HCM hard composite outcome consists of death, stroke, cardiac arrest, myectomy, LVAD implantation or cardiac transplantation. Major adverse cardiovascular events (MACE) consists of heart failure, atrial fibrillation, stroke or cardiac arrest. **(d)** Effect of PGS on CMR imaging traits in 440 HCM cases at the Royal Brompton Hospital, stratified by genetic status (101 SARC-PLP, 104 SARC-Neg) and all (440 cases, including 235 with VUS or unknown variant status). Data presented as standardized effect size for each SD increase in HCM PGS from a linear regression model with 95% confidence intervals.

Supplementary Figure References

1. Evangelou, E. *et al.* Genetic analysis of over 1 million people identifies 535 new loci associated with blood pressure traits. *Nat Genet* **50**, 1412-1425 (2018).
2. Chen, J. *et al.* The trans-ancestral genomic architecture of glycemic traits. *Nature Genetics* **53**, 840-860 (2021).
3. Willer, C.J. *et al.* Discovery and refinement of loci associated with lipid levels. *Nature Genetics* **45**, 1274-1283 (2013).
4. Elsworth, B. *et al.* The MRC IEU OpenGWAS data infrastructure. *bioRxiv*, 2020.08.10.244293 (2020).
5. Shah, S. *et al.* Genome-wide association and Mendelian randomisation analysis provide insights into the pathogenesis of heart failure. *Nat Commun* **11**, 163 (2020).
6. Nielsen, J.B. *et al.* Biobank-driven genomic discovery yields new insight into atrial fibrillation biology. *Nature Genetics* **50**, 1234-1239 (2018).

LV trait GWAS and multi trait analysis of GWAS

Multi-trait analysis of GWAS was performed for HCM using genetically correlated traits as previously described[1]. GWAS of ten left ventricular (LV) cardiac magnetic resonance (CMR) phenotypes was performed in 39,559 participants in the imaging substudy of UK Biobank (UKB) as previously described[1]. Individuals with ICD-10 code and self-reported diagnoses of heart failure, cardiomyopathy, previous myocardial infarction, or structural heart disease were excluded. After imaging and genotype quality control, exclusion of comorbidities, and restricting to a subset of unrelated individuals (3rd degree or closer), there was a maximum cohort size of 36,083 individuals. CMR imaging analysis has previously been described[1,2]. The ten included LV phenotypes for GWAS included LV end diastolic volume (LVEDV), end-systolic volume (LVESV), ejection fraction (LVEF), mass (LVM), concentricity index (LVconc = LVM/LVEDV), mean wall thickness (meanWT), maximum wall thickness (maxWT) and global peak strain in radial, longitudinal and circumferential directions. The GWAS model was adjusted for age, sex, mean arterial pressure, body surface area, and the first 8 genetic principal components.

Selection of traits to be incorporated with HCM in MTAG was limited to 4 traits (HCM and 3 LV traits) due to computational burden of computing the maximum false discovery rate (maxFDR). Selection of the 3 LV traits was performed as follows. First, pairwise genetic correlation between LV traits (including HCM) was assessed using LD score regression (LDSC v1.1.1)[3] using precomputed LD scores from the European 1000 Genomes Project. Second, heirarchical clustering of the 10 LV traits using the absolute value of the pairwise genetic correlations, Euclidean distance and the complete method, predefining the number of clusters to 3. This resulted in clustering of LV traits into an LV contractility cluster (LVEF, global peak strain in radial longitudinal, and circumferential direction), an LV volume cluster (LVEDV, LVESV) and an LV mass cluster (LVM, LVconc, meanWT, and maxWT). Finally, we selected the trait with the highest genetic correlation with HCM from each cluster to include in MTAG together with HCM (Figure).

MTAG was performed using *mtag* using default settings[4]. MTAG can result in inflation of results and false positives when there is poor genetic correlation or when GWAS of associated traits have greater power than the GWAS of interest (in this case HCM). The maxFDR calculates the type I error in the analyzed dataset for the worst-case scenario, and was calculated as suggested by the MTAG developers[4].

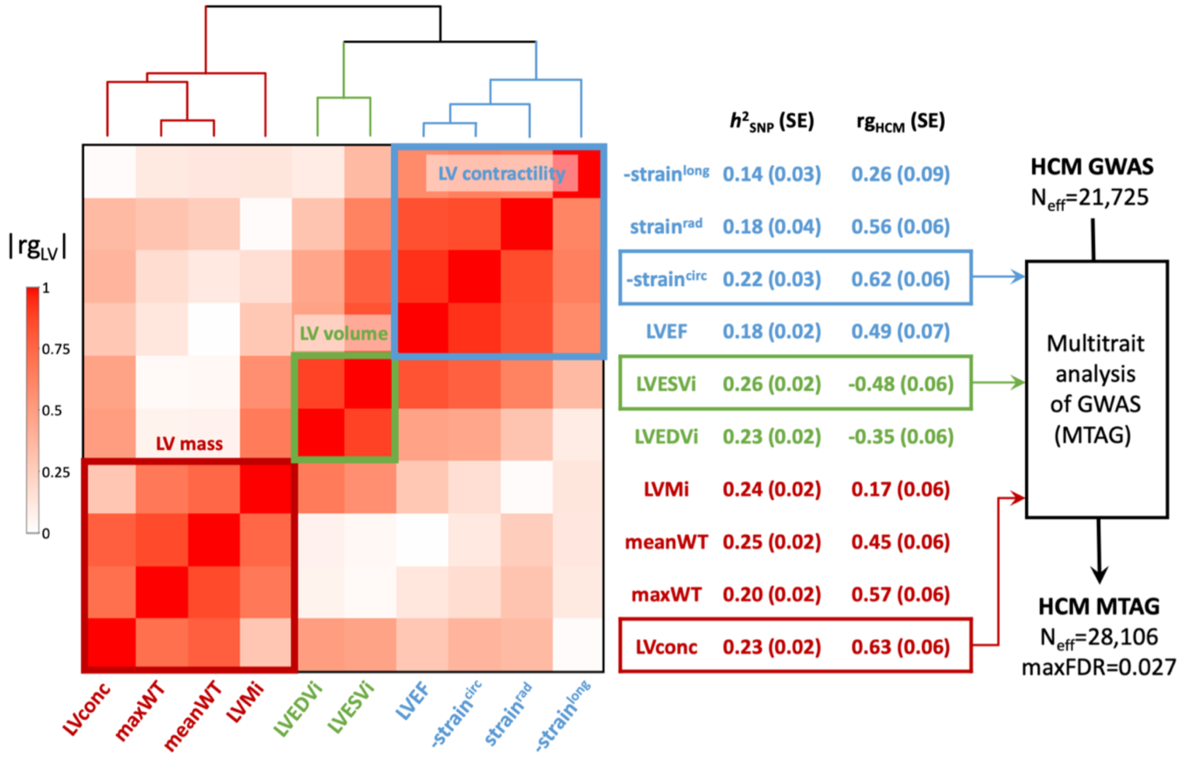

Figure (from Tadros et al.[1]) showing the genetic correlation (rg_{HCM}) and heirarchical clustering of LV traits with HCM. LV traits included with HCM in MTAG were selected as the trait with the highest correlation with HCM (rg_{HCM}) from each of the 3 clusters representing LV mass (red), volumetric (green), and contractility (blue). MTAG improved discovery power, with an increase in effective GWAS number from 21,725 to 28,106. The maximum directly computed false discovery rate was 0.027.

HCM definition across cohorts

UK Biobank

- Self-reported hypertrophic cardiomyopathy (HCM) or hypertrophic obstructive cardiomyopathy (HOCM) at time of study enrollment (UKB field 20002) in the absence of aortic stenosis.
- ICD-10 (I42.1, I42.2) or ICD-9 (425.1) code for HCM or HOCM recorded on hospital episode statistic or on death record (https://github.com/UK-Digital-Heart-Project/UKBB_filter), in the absence of aortic stenosis (UKB fields 40001, 40002, 41202, 41204, and 41270).
- Maximum LV wall thickness of at least 15mm in the absence of aortic stenosis with SBP<140mmHg on date of scan, quantified using approach in *De Marvao et al.*[5]

100K Genomes Project

- Referral to study with diagnosis of HCM or HOCM from HPO terms.
- ICD-10 (I42.1, I42.2) code for HCM or HOCM recorded on hospital episode statistic.

Erasmus Medical Centre, NL

- Clinical diagnosis of HCM/HOCM using guideline-based criteria (imaging, personal and family history, examination, genetic, and other investigations)[6,7].

Royal Brompton Hospital, UK

- Clinical diagnosis of HCM/HOCM using guideline-based criteria (imaging, personal and family history, examination, genetic, and other investigations)[6,7].

National Heart Center Singapore, Singapore

- Clinical diagnosis of HCM/HOCM using guideline-based criteria (imaging, personal and family history, examination, genetic, and other investigations)[6,7].

Clinical outcomes in UK Biobank

HCM composite:
- Death
- HCM
- Heart failure
- Atrial fibrillation/flutter
- Stroke
- Cardiac arrest
- Septal reduction therapy (surgical myectomy or alcohol septal ablation)
- ICD implantation
- LVAD implantation
- Cardiac transplantation

HCM hard composite:
- Death
- Stroke
- Cardiac arrest
- Surgical myectomy
- LVAD implantation
- Cardiac transplantation

Major adverse cardiovascular:
- HCM
- Heart failure
- Atrial fibrillation/flutter
- Stroke
- Cardiac arrest

Note: for analysis of clinical outcomes in HCM cases, HCM diagnosis was excluded from all of the above composite outcomes.

Erasmus Medical Centre cohort methods

Clinical outcomes

Three endpoints were predefined and evaluated in the Erasmus Medical Centre (EMC) cohort of 214 sarcomere-positive relatives of HCM probands.

1. HCM diagnosis
2. LV maximum wall thickness (maxLVWT) at last available transthoracic echocardiogram (TTE) or cardiac magnetic resonance imaging (CMR). For participants who underwent cardiac transplantation and/or septal reduction therapy, the last available imaging prior to cardiac transplantation and/or septal reduction therapy was used. Given the higher precision of CMR to assess wall thickness, maxLVWT from CMR was used whenever available unless CMR performed more than 5 years before the most recent TTE.
3. Time to first major adverse clinical event, a composite of myectomy therapy, alcohol ablation, cardiac transplantation, sustained ventricular arrhythmia, sudden cardiac death, or appropriate defibrillator shock.

Statistical analysis

The association between PGS_{HCM} and HCM was assessed using a Wald logistic mixed-effects model using the *glmm.wald()* function from R-package GMMAT (v.1.3.2)[8], adjusting for fixed-effects of sex, age, age^2 and PCs 1-4. To account for the between-sample relatedness, we incorporated a genetic relatedness matrix (GRM), estimated using GCTA (v.1.92.4 beta)[9], as a random effect. The association of PGS_{HCM} with maxLVWT was assessed using a linear mixed-effects model implemented in function *lmekin()* from R-package coxme (v.2.2-17), adjusting for sex, age at imaging, age at $imaging^2$, imaging modality (CMR vs TTE), ancestral PCs 1 to 4, and the GRM. The association of PGS_{HCM} with incident major clinical events was assessed using a Cox proportional hazards mixed-effects model using function *coxme()* from R-package coxme. Time 0 was set to birth in the Cox model and study participants were censored at the time of last clinical follow up. Sex, ancestral PCs 1 to 4 and the GRM were again included as covariates. *MYH7* rare variant genotype was more prevalent among samples with higher PGS_{HCM} (**Supplementary Table 1**); despite rare variant genotype not being found associated with any of the three endpoints, we performed sensitivity analyses adjusting for rare variant gene for each endpoint.

Primary analyses were performed using PGS_{HCM} as a continuous variable. In secondary analyses, we split PGS_{HCM} into quintiles and performed analyses testing top PGS quintile versus bottom PGS quintile, and top quintile versus median quintile. These analyses were performed to assess effect sizes between meaningful extremes of the PGS distribution.

Variant pathogenicity

UK Biobank
Pathogenic rare variants in 8 HCM-causing genes (*MYBPC3, MYH7, TNNT2, TNNI3, TPM1, ACTC1, MYL3,* and *MYL2*) were identified from whole exome sequencing data using the following approach[5]. Variants 100bp up or downstream of genes with a minor allele frequency of <0.1% in gnomAD and UKB were extracted. Splice region variants reported as pathogenic in ClinVar were manually curated for functional evidence of splicing[10]. LOFTEE[11] was used to identify low confidence predicted loss of function (pLOF) variants, and all remaining pLOF variants were annotated for prediction of nonsense mediated decay (NMD) escape. The variants were then filtered for disease-causing mechanism and met a filtering allele frequency of <0.00004 in gnomAD: all MYBPC3 protein-altering variants were kept, and for the other 7 genes only variants influencing gene product structure or level were retained. Finally, the variant list was shorted to only include variants that would be called pathogenic or likely pathogenic in a patient with HCM using CardioClassifier[12] and ClinVar, with manual curation of variants if they had evidence of pathogenicity.

100K Genomes Project
Pathogenic rare variants in 8 HCM-causing genes (*MYBPC3, MYH7, TNNT2, TNNI3, TPM1, ACTC1, MYL3,* and *MYL2*) were identified from whole genome sequencing data. Variants 100bp up or downstream of genes with a minor allele frequency of <0.1% in gnomAD and GeL were filtered for pathogenicity using ClinVar (annotated as pathogenic or likely pathogenic for HCM from multiple submitters without conflict) or CardioClassifier[12], and for disease-causing mechanism (variants influencing gene product structure or level in *MYH7, TNNT2, TNNI3, TPM1, ACTC1, MYL3,* and *MYL2*).

Royal Brompton Hospital
HCM probands were sequenced using the Illumina Trusight Cardio Sequencing Kit on Illumina MiSeq and NextSeq platforms and a custom Agilent SureSelect cardiac gene panel on the Life Technologies SOLiD 5500xl platform. Rare variants were defined as having a mean allelic frequency of less than $1x10^{-4}$ in ExAC, and were required to be protein-altering (missense, nonsense, frameshift, in-frame indels, and essential splice site) and with high quality calling (PASS filter). Rare variant pathogenicity was determined following ACMG variant classification guidelines[13,14] and incorporated the use of the CardioClassifier resource[12].

Erasmus Cohort
Apart from genotyping using the Global Screening Array (in the research setting), all Erasmus cohort samples also underwent sequencing or targeted genotyping in the context of clinical genetics assessments. All samples included in the present study were found to be carriers of class 4 or class 5 variants in sarcomere genes (*MYBPC3*, *MYH7, TNNT2, TNNI3, TPM1, ACTC1, MYL3,* and *MYL2*). Variant pathogenicity was assessed centrally according to the American College of Medical Genetics and Genomics and the Association for Molecular Pathology (ACMG/AMP) guidelines[13] using an adapted version of the CardioClassifier resource[12], as described previously[2]. Homozygous carriers and those carrying multiple pathogenic or likely pathogenic variants were excluded in the present analysis."

HCM GWAS collaborators

Rafik Tadros, Sean L Zheng, Christopher Grace, Paloma Jordà, Catherine Francis, Sean J Jurgens, Kate L Thomson, Andrew R Harper, Elizabeth Ormondroyd, Dominique M West, Xiao Xu, Pantazis I Theotokis, Rachel J Buchan, Kathryn A McGurk, Francesco Mazzarotto, Beatrice Boschi, Elisabetta Pelo, Michael Lee, Michela Noseda, Amanda Varnava, Alexa MC Vermeer, Roddy Walsh, Ahmad S Amin, Marjon A van Slegtenhorst, Nicole Roslin, Lisa J Strug, Erika Salvi, Chiara Lanzani, Antonio de Marvao, Hypergenes InterOmics Collaborators, Jason D Roberts, Maxime Tremblay-Gravel, Genevieve Giraldeau, Julia Cadrin- Tourigny, Philippe L L'Allier, Patrick Garceau, Mario Talajic, Yigal M Pinto, Harry Rakowski, Antonis Pantazis, John Baksi, Brian P Halliday, Sanjay K Prasad, Paul JR Barton, Declan P O'Regan, Stuart A Cook, Rudolf A de Boer, Imke Christiaans, Michelle Michels, Christopher M Kramer, Carolyn Y Ho, Stefan Neubauer, HCMR Investigators, Paul M Matthews, Arthur A Wilde, Jean-Claude Tardif, Iacopo Olivotto, Arnon Adler, Anuj Goel, James S Ware , Connie R Bezzina, Hugh Watkins

Supplementary Note references

1. Tadros, R., *et al.* Large scale genome-wide association analyses identify novel genetic loci and mechanisms in hypertrophic cardiomyopathy. *medRxiv*, 2023.2001.2028.23285147 (2023).
2. Tadros, R., *et al.* Shared genetic pathways contribute to risk of hypertrophic and dilated cardiomyopathies with opposite directions of effect. *Nature Genetics* **53**, 128-134 (2021).
3. Bulik-Sullivan, B., *et al.* An atlas of genetic correlations across human diseases and traits. *Nat Genet* **47**, 1236-1241 (2015).
4. Turley, P., *et al.* Multi-trait analysis of genome-wide association summary statistics using MTAG. *Nat Genet* **50**, 229-237 (2018).
5. de Marvao, A., *et al.* Phenotypic Expression and Outcomes in Individuals With Rare Genetic Variants of Hypertrophic Cardiomyopathy. *J Am Coll Cardiol* **78**, 1097-1110 (2021).
6. Elliott, P.M., *et al.* 2014 ESC Guidelines on diagnosis and management of hypertrophic cardiomyopathy: the Task Force for the Diagnosis and Management of Hypertrophic Cardiomyopathy of the European Society of Cardiology (ESC). *Eur Heart J* **35**, 2733-2779 (2014).
7. Ommen, S.R., *et al.* 2020 AHA/ACC Guideline for the Diagnosis and Treatment of Patients With Hypertrophic Cardiomyopathy: Executive Summary: A Report of the American College of Cardiology/American Heart Association Joint Committee on Clinical Practice Guidelines. *Circulation* **142**, e533-e557 (2020).
8. Chen, H., *et al.* Control for Population Structure and Relatedness for Binary Traits in Genetic Association Studies via Logistic Mixed Models. *The American Journal of Human Genetics* **98**, 653-666 (2016).
9. Yang, J., Lee, S.H., Goddard, M.E. & Visscher, P.M. GCTA: a tool for genome-wide complex trait analysis. *Am J Hum Genet* **88**, 76-82 (2011).
10. Jaganathan, K., *et al.* Predicting Splicing from Primary Sequence with Deep Learning. *Cell* **176**, 535-548.e524 (2019).
11. Karczewski, K.J., *et al.* The mutational constraint spectrum quantified from variation in 141,456 humans. *Nature* **581**, 434-443 (2020).
12. Whiffin, N., *et al.* CardioClassifier: disease- and gene-specific computational decision support for clinical genome interpretation. *Genetics in Medicine* **20**, 1246-1254 (2018).
13. Richards, S., *et al.* Standards and guidelines for the interpretation of sequence variants: a joint consensus recommendation of the American College of Medical Genetics and Genomics and the Association for Molecular Pathology. *Genet Med* **17**, 405-424 (2015).
14. Walsh, R., *et al.* Reassessment of Mendelian gene pathogenicity using 7,855 cardiomyopathy cases and 60,706 reference samples. *Genet Med* **19**, 192-203 (2017).
