# Supplementary material for: Evaluation of polygenic scores for hypertrophic cardiomyopathy in the general population and across clinical settings
Source: Nat Genet. 2025 Feb 18;57(3):563–71. doi: 10.1038/s41588-025-02094-5 (PMC11906360; doi:10.1038/s41588-025-02094-5)
Supplement: Supplementary file 2 — Reporting Summary [file 41588_2025_2094_MOESM2_ESM.pdf]

nature portfolio

Corresponding author(s): James Ware
Last updated by author(s): 25-04-2023

Reporting Summary

Nature Portfolio wishes to improve the reproducibility of the work that we publish. This form provides structure for consistency and transparency in reporting. For further information on Nature Portfolio policies, see our Editorial Policies and the Editorial Policy Checklist.

Statistics

For all statistical analyses, confirm that the following items are present in the figure legend, table legend, main text, or Methods section.

n/a	Confirmed	
☐	☒	The exact sample size (*n*) for each experimental group/condition, given as a discrete number and unit of measurement
☐	☒	A statement on whether measurements were taken from distinct samples or whether the same sample was measured repeatedly
☐	☒	The statistical test(s) used AND whether they are one- or two-sided<br>*Only common tests should be described solely by name; describe more complex techniques in the Methods section.*
☐	☒	A description of all covariates tested
☐	☒	A description of any assumptions or corrections, such as tests of normality and adjustment for multiple comparisons
☐	☒	A full description of the statistical parameters including central tendency (e.g. means) or other basic estimates (e.g. regression coefficient) AND variation (e.g. standard deviation) or associated estimates of uncertainty (e.g. confidence intervals)
☐	☒	For null hypothesis testing, the test statistic (e.g. *F*, *t*, *r*) with confidence intervals, effect sizes, degrees of freedom and *P* value noted<br>*Give P values as exact values whenever suitable.*
☒	☐	For Bayesian analysis, information on the choice of priors and Markov chain Monte Carlo settings
☒	☐	For hierarchical and complex designs, identification of the appropriate level for tests and full reporting of outcomes
☐	☒	Estimates of effect sizes (e.g. Cohen's *d*, Pearson's *r*), indicating how they were calculated

Our web collection on statistics for biologists contains articles on many of the points above.

Software and code

Policy information about availability of computer code

Data collection	No software was used for data collection.
Data analysis	PGS was generated from GWAS summary statistics using PRS-CS v1.0 (https://github.com/getian107/PRScs) and PRS-CSx v1.0 (https://github.com/ getian107/PRScsx). PGS scores for individuals were calculated using PLINK version 1.9. Statistical analysis was performed using R version 4.0.3. PGS model performance was assessed using pROC v1.18.0 package in R. Association testing between PGS and outcomes in Erasmus Medical Centre Cohort was performed using GMMAT v1.3.2 and coxme v2.2-17 packages in R, with a genetic relatedness matrix calculated using GCTA v1.92.2b). Survival analysis was performed using survival v3.5-7 and survminer v0.4.9 in R. Singapore genotyping data was imputed using Minimac4 (v1.5.7) and GWAS was performed using SNPTEST version 2.5.6. PheWAS was performed using PheWAS (v0.99.5-5) in R. Two-sample Mendelian randomisation was performed using TwoSampleMR (v0.6.4) and MRInstruments (v0.3.2) in R. R version 4.0.3 was used. Custom analysis code is available from Zenodo (https://zenodo.org/records/11204463).

For manuscripts utilizing custom algorithms or software that are central to the research but not yet described in published literature, software must be made available to editors and reviewers. We strongly encourage code deposition in a community repository (e.g. GitHub). See the Nature Portfolio guidelines for submitting code & software for further information.

Data

Policy information about availability of data

All manuscripts must include a data availability statement. This statement should provide the following information, where applicable:

- Accession codes, unique identifiers, or web links for publicly available datasets
- A description of any restrictions on data availability
- For clinical datasets or third party data, please ensure that the statement adheres to our policy

Data from UK Biobank can be requested from the UK Biobank Access Management System (https://www.ukbiobank.ac.uk/enable-your-research/apply-for-access). Data from 100,000 Genomes Project can be accessed following application to join the Genomics England Clinical Interpretation Partnership (https://www.genomicsengland.co.uk/research/academic/join-research-network). The PGS are available for download from the Polygenic Score Catalog (https://www.pgscatalog.org) under accession IDs PGS004910 and PGS004911. GWAS and MTAG results10 used to generate PGS are available for download from the GWAS Catalog (https://www.gwascatalog.org) under accession IDS GCST90432127 and GCST904321230.

Human research participants

Policy information about studies involving human research participants and Sex and Gender in Research.

Reporting on sex and gender	The article uses the term sex when referring to biological attribute, and was determined using genetic sex where available. Sex was included as a covariate in all multivariate analyses. Findings are relevant to both male and females.
Population characteristics	Population characteristics include age, sex, ancestry (self-reported and genetic) and genetic principal components for all individuals. Blood pressure and body surface area was available for individuals in the cardiac magnetic resonance imaging substudy of the UK Biobank.
Recruitment	Participants were recruited to the UK Biobank from a large number of national sources (e.g. GP, leaflets and advertising, hospitals, and recruitment drives in the community), and targeted individuals from middle age onwards. This results in the enrichment of less penetrant variants. 100,000 Genomes Project recruited patients with rare disease and cancer along with their relatives, from clinical centres, initially with an emphasis on genetically unexplained disease. This results in enrichment of individuals with sarcomere-negative HCM. HCM cases were recruited from the Royal Brompton Hospital, Erasmus Medical Center and National Heart Center Singapore directly from clinics.
Ethics oversight	All patients gave written informed consent, and all studies were approved by the relevant regional research ethics committees, and adhered to the principles set out in the Declaration of Helsinki. The UK Biobank study was reviewed by the National Research Ethics Service (11/NW/0382, 21/NW/0157). The 100,000 Genomes Project was reviewed by the National Research Ethics Service (14/EE/1112 and 13/EE/032). The Royal Brompton Biobank was reviewed and approved by the South Central – Hampshire B Research Ethics Committee (09/H0504/104+5 and 19/SC/0257). The Erasmus Medical Center was reviewed and approved by the Erasmus MC Medical Ethical Review Committee. All Singaporean participants recruited from the National Heart Center Singapore gave written informed consent and the study was approved by the Singhealth Centralised Institutional Review Board (2020/2353) and the Singhealth Biobank Research Scientific Advisory Executive Committee (SBRSA 2019/001v1).

Note that full information on the approval of the study protocol must also be provided in the manuscript.

Field-specific reporting

Please select the one below that is the best fit for your research. If you are not sure, read the appropriate sections before making your selection.

☒ Life sciences ☐ Behavioural & social sciences ☐ Ecological, evolutionary & environmental sciences

For a reference copy of the document with all sections, see nature.com/documents/nr-reporting-summary-flat.pdf

Life sciences study design

All studies must disclose on these points even when the disclosure is negative.

Sample size	The polygenic score was initially evaluated in the UK Biobank, a population-based cohort with >500K individuals. For additional analyses, we used all available cases where possible and made efforts to maximise case numbers where possible.
Data exclusions	No data were excluded from the analysis.
Replication	Polygenic scores were generated using a Bayesian approach (PRS-CS) that negates the need for replication. Nonetheless, we ensured robustness and generalizability of our findings by testing the effects of PGS in several cohorts - including UK Biobank, 100,000 Genomes Project, and 2 clinical cohorts (Royal Brompton and Harefield NHS Foundation Trust, and Erasmus Medical Center).
Randomization	Observational study - not applicable

Blinding	Observational study - not applicable

Reporting for specific materials, systems and methods

We require information from authors about some types of materials, experimental systems and methods used in many studies. Here, indicate whether each material, system or method listed is relevant to your study. If you are not sure if a list item applies to your research, read the appropriate section before selecting a response.

Materials & experimental systems

n/a	Involved in the study
☒	☐ Antibodies
☒	☐ Eukaryotic cell lines
☒	☐ Palaeontology and archaeology
☒	☐ Animals and other organisms
☒	☐ Clinical data
☒	☐ Dual use research of concern

Methods

n/a	Involved in the study
☒	☐ ChIP-seq
☒	☐ Flow cytometry
☒	☐ MRI-based neuroimaging
